# A multi-proxy bioarchaeological approach reveals new trends in Bronze Age diet in Italy

**DOI:** 10.1038/s41598-022-15581-0

**Published:** 2022-07-16

**Authors:** Alessandra Varalli, Jacopo Moggi-Cecchi, Gwenaëlle Goude

**Affiliations:** 1 Aix Marseille Univ, CNRS, Minist Culture, LAMPEA, Aix-en-Provence, France; 2 Department of Biology, Laboratory of Anthropology, University of Florence, Florence, Italy; 3 CaSEs Research Group, Department of Humanities, Universitat Pompeu Fabra, Barcelona, Spain

**Keywords:** Archaeology, Biological anthropology

## Abstract

This study investigates changes in dietary practices and subsistence strategies in Bronze Age Italy integrating isotopic analyses with archaeobotanical and archaeozoological data. By investigating food habits, we contribute to reconstructing human lifestyles and highlighting possible links with the economic/social organization when the rise of stratified societies and new economic activities affected subsistence practices. Stable isotopes analyses in humans and animals were performed on 6 Italian sites dating from 2300 to 900 cal. BC, followed by a complete review of additional 19 sites, which forms the basis of a diachronic and geographic comparison for Bronze Age Italy. The geographic analysis shows a more varied diet in northern and central Italy, compared to the south. The diachronic analysis highlights the homogeneity of food habits during the Early Bronze Age, contrary to the later phases when an increase in dietary variability and a higher animal protein consumption are revealed. The Middle Bronze Age appears as a pivotal moment in protohistoric societies, a phase of transition. The consumption of different foodstuffs highlights the importance of cultural exchanges, resulting in a sort of “food globalization”, although environmental and climatic fluctuations could also have affected dietary patterns, favoring some crops over others.

## Introduction

The Bronze Age in Europe is a cultural conundrum. According to Heyd^1^, it lies between a supra-regional cultural phenomenon and very local declinations of the same phenomenon. With regional pottery styles and metallurgy dominating the archaeological debate, less attention has been paid to other cultural practices such as subsistence strategies, with the general agreement that subsistence in Bronze Age Europe relied on neolithic plants and animals which would have been exploited in a almost unaltered combination^2,3^. Any speculation on the Bronze Age diet would be made as part of a reflection of social complexity in the food regime, in association either with age/gender or, more generally, social inequalities^4^. For the Bronze Age, more often than for earlier periods, food has been investigated for its ability to mark distinctions or to convey political/social agreement^5^.

Many of the studies on Italian subsistence strategies for late prehistoric contexts have shown considerable regional variations. However, any attempt to explore differences among social categories has only revealed scattered results. Furthermore, one of the main problems is that most of these studies focus mainly on a single proxy, e.g. archaeobotanical evidence, providing only a partial view of dietary trends.

The aim of this research is to reconstruct Bronze Age dietary behavior as well as detect possible changes in the human subsistence economy putting together the growing archaeozoological, archaeobotanical and isotopic literature on prehistoric Italy. This is possible through an integrated approach examining new and existing data from a broader perspective. Specifically, this paper aims to present a comprehensive overview of Italian Bronze Age human diets, bringing together a set of published isotopic records and new data, in order to reconstruct geographical variation within the Italian Peninsula and diachronic changes during the Bronze Age. The possibility that environmental constraints would have driven local variation and regional differences and the link between cultural traditions and local ecosystems, will also be explored and evaluated.

## Synopsy of the subsistence strategies of the Italian Bronze Age

### The archaeobotanical evidence

The most common plant species exploited during the Italian Bronze Age are barley (*Hordeum vulgare*), einkorn wheat (*Triticum monococcum*) and emmer wheat (*Triticum dicoccum*), which belong to the so-called northeast Neolithic package^6–8^. Significant changes occurred between the Neolithic and the Bronze Age: the spread of “minor” plant species has been recorded between the end of the 3rd and the beginning of the 2nd millennium BC^9^. Although the chronology of these changes is still not clear, the origin of this phenomenon seems to be a consequence of the necessity to overcome climatic fluctuations, as well as new social practices as attested from the second half of the 3rd millennium, as suggested in other Eurasia areas^10^. Likely linked to an increase in demand due to the raise of the population size, new technologies, like irrigation, crop rotation, manuring and specific agronomic strategies, were introduced in continuity with regional agricultural systems both in the Alpine and in the southern regions like Apulia, where most of the archaeobotanical southern Italy investigations took place^11–15^. It is from this point on that the spectrum of cereal and common edible plants becomes more diverse. The northeast traditional cereals were still cultivated, but in addition to these, species such as broomcorn millet (*Panicum miliaceum*), foxtail millet (*Setaria italica*), rye (*Secale cereale*), spelt (*Triticum spelta*), *Avena* sp. and legumes (e.g., *Pisum sativum, Vicia ervilia, Lens culinaris*) spread over the peninsula^6,16,17^.

Crops such as oats and rye are documented as cultivated only in more recent periods (Iron Age)^6^. Hence, such findings during the Bronze Age have been identified as weeds^6,18,19^. The limited botanical record does not shed light on the importance of these plants in people’s livelihoods. The major findings are from the south of the peninsula, whereas the finds from the northern areas suggest these plants were mainly weeds. As for spelt, consistent evidence for its cultivation has only been recorded from the beginning of the Bronze Age. Spelt has been found at different sites in northern Italy, but its distribution is not yet well defined. Its presence in Trentino Alto Adige is probably due not only to its hardiness but also to the influence of Nordic countries, as this cereal seems to be much more widespread in central Europe than the Italian Peninsula^20^.

Particular attention must be devoted to millets, namely broomcorn and foxtail millet. The growing importance of these species during Later Prehistory calls for a critical reappraisal. An ever increasing number of botanical and isotopic discoveries all over Europe underscore the importance of these plants in human life^21^. Studies also highlight the spread of millets in Italy, supporting its substantial role in the human diet from prehistory to historical periods^22–26^.

Despite these findings, the spread of broomcorn and foxtail millet in Italy is still unclear. Identifying seeds from Neolithic sites is not always straightforward (e.g., Valgrana, Levata di Curtatone)^9,27^ and findings are often limited to a few seeds (e.g., Castello d’Annone, Capo Rondinella, San Domenico, Gr. della Tartaruga)^27,28^. Nevertheless, the archaeobotanical data from northern Italy suggest that broomcorn and foxtail millet were an important complement to cereal production during the Bronze Age^9,15^. The evidence is different for the center and the south of the peninsula, where botanical remains for these crops are rare^7,16^. Furthermore, archeobotanical studies are unable to determine whether a certain crop was for human or for domestic animal consumption. Conversely, isotopic analyses of human and animal samples are a useful tool in identifying millet intake, as plants with C_4_ photosynthesis, like millets, can be clearly distinguished from other cereals that belong to the C_3_ group. Indeed, stable isotope analysis provides relevant information on the distribution and consumption of millets and at the same time paves the way for new questions about the dynamics of the evolution of farming practices. It is in this context that research conducted in Italy during such a critical period is essential to explore the introduction of these species into the Mediterranean area.

Another C_4_ plant that occasionally played a significant role in Italy is the cockspur grass (*Echinochloa crus-galli*). Less widespread than broomcorn and foxtail millet in archaeological contexts, its ecological characteristics are similar to those of broomcorn and foxtail millet, except that it is better suited to wet environments. Substantial amounts of *Echinochloa* have been recorded for Bronze Age sites in northern Italy, Solarolo and Castellaro di Vho^9,19^. At present, there is no evidence of its presence in central and southern Italy^16^.

## Animal husbandry

Research on animal husbandry in Bronze Age Italy reveals the existence of diversified settings^28–31^. The different distribution of domestic species depends on geographical and environmental physiognomy and traditional local models responsible for diversified fauna management strategies^29,30–36^.

At present, it is possible to draw a diachronical picture of animal husbandry for the different phases of the Bronze Age only for a few sites^32^. However, some general trends can be outlined. First, from the MBA there was a general increase in the exploitation of secondary products from domestic mammals^30^ leading to more sedentary animal breeding^33^. Secondly, hunting became increasingly marginal and cattle were predominantly slaughtered in adulthood during the Bronze Age^37^.

Animal husbandry practices in northern Italy during the Bronze Age were varied. Within this marked heterogeneity, cattle seem to experience the greatest variations in percentage ratios. They range from 10% in Cattolica^38^ to 50–60% in Sonnenburg^39^, compared to the slightly more stable percentages for sheep, goats and pigs^34^. In the Alpine area, there was a general abundance of caprines (sheep and goats) and a shortage of pigs^36^. In the South Tyrolean and Trentino sites, sheep were preferentially selected as livestock because of the geography, e.g. Sotćiastel^35,40^. Nevertheless, cattle represented a significant component of the local husbandry economy as well, e.g. Val Pusteria^29,32,33,35,39,41^. In the Garda regions, there appears to have been greater variability in term of subsistence strategies (agriculture, livestock, harvesting of wild fruits, fishing, hunting) thanks to the heterogeneity of the available resources. Indeed, this area appears to be the only one for which caprines, cattle and pigs are equally represented^41^. In the area north of the Po River, and particularly that occupied by pile-dwellings, all the domestic species are well represented. To a limited extent, cattle or sheep and goats breeding dominated in some areas where pigs were less prominent than in the subalpine and Garda areas^29,33,42^. Moreover, some settlements suggest that cattle breeding increased during the RBA^31–34^. South of the Po, where Terramare settlements spread, the economy was substantially pastoral as caprines were the main resource, such as in the pile-dwelling villages of Trentino^29,33,34,43^.

As for elsewhere in the peninsula, husbandry practices are less well-known, due to the limited number of studies.

In central Italy (Toscany and Lazio), husbandry was more focused on cattle, sheep and goats and less on pigs, e.g. Petrosa^44,45^. In this area at the beginning of the MBA, the animal economy was focused on the breeding of cattle, however from the late MBA husbandry strategies changed and caprines became predominant. This trend continued throughout the RBA and FBA up to the early Iron Age, when pigs become more significant^46^. During the last phases of the Bronze Age, pastoral activities seem to increase and caprines become the main species at the sites of Marche and Abruzzo^47^. Research in the Marche region during the MBA suggests a mixed type economy. Nevertheless, some settlements may have had a more pastoral economy, while others had subsistence strategies based on agricultural practices associated with cattle and pig husbandry^48^. For this region too, it seems that there was a marked increase in caprines mainly used for meat. Moreover, it seems that the surplus of sheep could be interpreted as a prestigious goods of the emerging elites^43^.

In southern Italy, studies on sites such as Broglio di Trebisacce^49^, Torre del Mordillo^50^ and Coppa Nevigata^51^ allow a diachronic analysis of of changes in the animal economy. However, the lack of information for the EBA constitutes a substantial gap^52^. Higher percentages of caprines compared to cattle and pigs have also been observed in southern Italy for the MBA and RBA where sheep were predominant^43,52^. Even though they were mainly exploited for meat, secondary products were important for the local economy, as recorded in Puglia and Sibaritide^49,50^. Notwithstanding, the sites with high percentages of cattle are numerous, e.g. Torre dei Passeri^53^, Monopoli^54^, and Bari S. Maria del Buon Consiglio^55^. These animals were no longer being reared exclusively for meat and other species such as sheep or pig also provided good quality meat. Occasionally game, i.e. deer, may also have purposefully supplemented meat production^37^. The increase of the number of calves in the animal records suggests an evolution of the dairy industry and the general prosperity of settlements^43^. In some of the southern sites, hunting was still significant for the local economy^54,56^ and seems to have intensified at the end of the Bronze Age. This phenomenon could be linked to increased demands for meat following a rise in the exploitation of secondary products^43^.

Evidence for aquatic species is rare for the Bronze Age sites of the Peninsula. When present, mainly freshwater and euryaline species are found in the north and in central Italy^57^. Pike and turtle have been found in the Lagazzi pile-dwellings and fishing is attested by tench, pike and rudd remains at Canàr^57,58^. Dating to the FBA and early IA, abundant remains of tench and pike have been found in Frattesina^57^. In the South of the Peninsula, the aquatic remains are mainly of saltwater species^56^ and the main discoveries are from the Apulia coast (Monopoli, Roca, Punta Le Terrare)^56^.

## The archaeological setting

**Figure 1:**
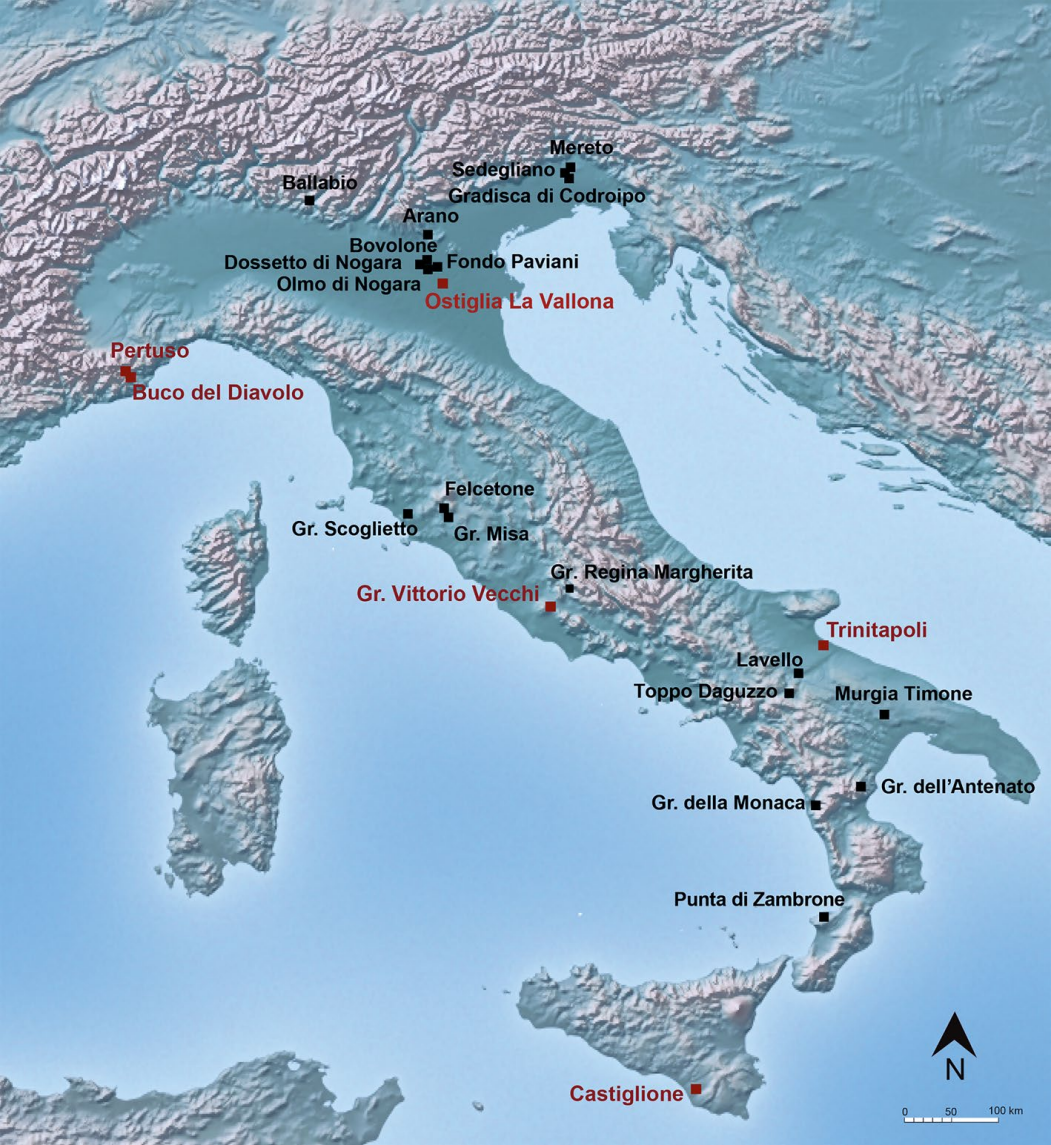
Location of the Italian Bronze Age cemeteries for which stable isotope analyses on humans are available (the sites from the literature are in black and the sites analysed here are in red). The map is elaborated using “Natural Earth. Free vector and raster map data @naturalearthdata.com” available at https://www.naturalearthdata.com/downloads/10m-raster-data).

The cemeteries of Pertuso, Buco del Diavolo, Ostiglia La Vallona, Gr. Vittorio Vecchi, Trinitapoli and Castiglione presented here for the first time are shown in Fig. 1 and Table 1. The selection of the sites was driven by the aim at increasing the representability of each chronological period and geographical area. It takes into account the limitations due to lesser explored areas, i.e. the center and south of the Peninsula, and the changes observed in funerary practices with the passage from inhumation to cremation during the MBA/FBA, which curb the analysis of bone collagen. The paucity and heterogeneity of the information provided by some of the newly discovered sites restricts this study, nevertheless they contribute substantially to the reconstruction of a dietary overview.

**Table 1:** Bronze Age Italian sites included in this overview. In bold the new sites presented in this study.

Site	Locality, Region	Altitude (a.s.l.)	Chronology^1^	Number	Reference (isotopic study)
**Pertuso (PER)**	**Imperia, Liguria**	**1330 m**	EBA-MBA*	**Humans, 14** **Animals, 14**	**(This study)**
**Buco del Diavolo (BdD)**	**Imperia, Liguria**	**1430 m**	FBA-EIA*	**Humans, 9** **Animals, 18**	**(This study)**
Ballabio (BAL)	Lecco, Lombardy	700 m	EBA-MBA*	Humans, 22 Animals, 3	^61^
**Ostiglia La Vallona (OLV)**	**Mantova, Lombardy**	**13 m**	**RBA**	**Humans, 19** **Animals, 4**	**(This study)**
Arano di Cellore (ARA)	Verona, Veneto	210 m	EBA*	Humans, 54 Animals, 13	^60^
Olmo di Nogara (OdN)	Verona, Veneto	18 m	MBA-RBA	Humans, 64 Animals, 5	^25,59^
Bovolone (BOV)	Verona, Veneto	24 m	MBA/RBA	Humans, 24 Animals, 0	^25^
Dossetto di Nogara (DOSS)	Verona, Veneto	20 m	EBA	Humans, 1 Animals, 3	^25^
Fondo Paviani (FP)	Verona, Veneto	16 m	MBA/RBA transitional phase-FBA	Humans, 0 Animals, 21	^25^
Mereto (ME)	Udine, Friuli Venezia Giulia	98 m	EBA	Humans, 1 Animals, 3	^25,59^
Sedegliano (SED)	Udine, Friuli Venezia Giulia	70 m	EBA-MBA	Humans, 2 Animals, 0	^59^
Gradisca di Codroipo (GC)	Udine, Friuli Venezia Giulia	36 m	RBA-FBA	Humans, 0 Animals, 3	^25^
Grotta dello Scoglietto (SCO)	Grosseto, Toscany	20 m	EBA	Humans, 11 Animals, 11	^26^
Grotta Misa (GM)	Viterbo, Lazio	138 m	MBA	Humans, 4 Animals, 4	^26^
Felcetone (FEL)	Viterbo, Lazio	200 m	MBA	Humans, 12 Animals, 0	^26^
**Grotta Vittorio Vecchi (GVV)**	**Latina, Lazio**	**505 m**	**MBA**	**Humans, 6** **Animals, 12**	**(This study)**
Grotta Regina Margherita (GRM)	Frosinone, Lazio	440 m	MBA*	Humans, 10 Animals, 13	^64^
Lavello (LAV)	Potenza, Basilicata	300 m	MBA	Humans, 4 Animals, 0	^59^
Toppo Daguzzo (TD)	Potenza, Basilicata	300 m	MBA	Humans, 21 Animals, 0	14 humans ^59^ 7 humans ^63^
Murgia Timone (MT)	Matera, Basilicata	425 m	BA	Humans, 0 Animals, 5	^63^
**Trinitapoli (TRI)** **(Ipogeo dei Bronzi)**	**Madonna di Loreto,** **Barletta-Andria-Trani, Apulia**	**10 m**	**MBA**	**Humans, 28** **Animals, 5**	**21 humans—this study** **7 humans** ^63^
**Castiglione (CAST)**	**Ragusa, Sicily**	**50 m**	**EBA**	**Humans, 96** **Animals, 0**	**(This study)**
Punta di Zambrone (PdZ)	Vibo Valentia, Calabria	220 m	RBA	Humans, 2 Animals, 15	^62^
Grotta della Monaca (GdM)	Cosenza, Calabria	740 m	MBA*	Humans, 6 Animals, 0	^63^
Grotta dell’Antenato (GdA)	Cosenza, Calabria	380 m	MBA*	Humans, 1 Animals, 0	^63^

^1^The chronology refers to radiocarbon dating (*). When absent, it refers to dating based on archaeological materials. Supplementary Information 1 for the relevant references.

*EBA* Early Bronze Age (ca. 2200–1650 BC), *MBA* Middle Bronze Age (ca. 1650–1350/1300 BC), *RBA* Recent Bronze Age (ca. 1350/1300–1150 BC), *FBA* Final Bronze Age (ca. 1150–950/925 BC).

The sites of Pertuso, Buco del Diavolo, Ostiglia La Vallona, Gr. Vittorio Vecchi, Trinitapoli and Castiglione, located in the north, center and south of the peninsula, span from EBA to the FBA/IA. These cemeteries include 164 humans and 51 domestic and wild animals recovered in association with the human remains, then they are all coeval. Each archaeological site context is detailed in Supplementary Text A.1.

These sites are compared with 19 sites previously investigated (Fig. 1, Table 1): Ballabio, Arano di Cellore, Mereto, Sedegliano, Gradisca di Codroipo, Dossetto di Nogara, Olmo di Nogara, Bovolone, Fondo Paviani, Gr. Misa, Gr. Scoglietto, Gr. Regina Margherita, Felcetone, Lavello, Toppo Daguzzo, Murgia Timone, Gr. della Monaca, P. di Zambrone and Gr. dell’Antenato^25,26,58–64^.

## Results

The carbon and nitrogen isotopic ratios from Pertuso, Buco del Diavolo, Ostiglia La Vallona, Gr. Vittorio Vecchi, Trinitapoli and Castiglione are presented in Supplementary Table A.1 and summarized in Supplementary Tables A.2 and A.3. The state of preservation is detailed in Supplementary Text A.2 and Table A.4.

To minimize the environmental differences, the humans have been compared considering the offset between the median of the humans and that of the associated domestic animals for each site to evaluate differences in the diets of human communities (Δ^13^C_h-f_ and Δ^15^N_h-f_). Indeed, considering that the diet–tissue trophic enrichment factor is estimated to be around 3–5‰ for the nitrogen and 0–1‰ for the carbon per trophic level^66,67^, the offsets between the human and fauna are good indicators of a community diet (protein intake).

The sites in Liguria, Pertuso and Buco del Diavolo, are dated to the EBA and FBA/EIA respectively. The two animal groups present typical ranges for a temperate environment predominantly dominated by C_3_ plants. When considering the herbivores of both sites, no significant differences occur for both δ^15^N ​​and δ^13^C values (δ^15^N, p = 0.361; δ^13^C, p = 0.854). This suggests that the two sites were placed in a similar environment and that no great environmental and/or paleoclimatic changes occurred during the Bronze Age in this area and that herbivores consumed similar plants. Since the two animal assemblages show the same food pattern, the two human groups are directly comparable. If any difference in dietary patterns appears between the two groups, it will likely be the result of food preferences rather than of a "different environmental offer".

### Pertuso (Liguria)

The δ^15^N human median is 7.2‰ (min = 6.5‰, max = 7.9 ‰, n = 14) and the δ^13^C median is − 20.6‰ (min = − 21.0‰, max = − 20.2‰, n = 14). The isotopic values show the consumption of a mixed terrestrial diet with resources typical of C_3_ plants environment. According to the Mediterranean marine resources isotopic data^68,69^, humans did not routinely consume seafoods. The human δ^15^N and δ^13^C results are spread in narrow ranges, suggesting a homogenous diet. Considering the δ^15^N and the δ^13^C human and domestic animal medians, the Δ^15^N_h-f_ and Δ^13^C_h-f_ are 3.5‰ and 0.2‰ respectively, values that are included in the recurring offsets between two different trophic levels. The Δ^15^N_h-f_ result indicates that the animal proteins played an important role in the diet of these individuals. When considering adults (n = 8) and juveniles (n = 4), significant statistical differences emerge for both δ^15^N (p = 0.048) and δ^13^C (p = 0.016). The juveniles show lower δ^15^N and δ^13^C values compared to the adults. A lower animal protein intake, possibly associated with vitamin and mineral deficiencies, may have contributed to weakening the youngest individuals, making them more vulnerable and inclined to disease and death, as supported by pathological evidence as well (Harris lines)^70^. Paleopathological analyses have also shown that adults present stress marks linked to their growth, possibly related to food deficiencies as well (dental hypoplasia, *cribra cranii* and *orbitalia*)^70^. All evidence seems to support that this community was exposed to repeated stress, especially during childhood. Similar cases in which the δ^15^N and δ^13^C results of juveniles are lower than for the rest of the population have been recorded in other sites in Liguria and in southeastern France for the Neolithic^71,72^.

Studies conducted on western Ligurian attest that Bronze Age communities were composed of small family groups^73^. Osteological studies on Pertuso humans seem to confirm this hypothesis, as olecranon perforations have been recorded in a number of individuals, supporting a certain degree of genetic links. The main settlements were in all likelihood scattered in naturally defended positions or consisted of fortified sites which controlled the surrounding area, e.g., Castellaro di Uscio^74^. As such, the archaeological evidence indicates an economy based on livestock and on the available local resources, as highlighted by the hunting, i.e. deer and wild boar, and gathering, i.e., apples, horns, acorns, that certainly enhances the evidence for a undifferentiated diet^73^.

### Buco del Diavolo (Liguria)

The δ^15^N and δ^13^C human results show the presence of two groups with different dietary patterns. The first group, represented by BDA, BD716, BD1320, BD1092 and BD2453, has a median of 7.2‰ for δ^15^N (min = 6.6‰, max = 7.5 ‰, n = 5) and − 19.8‰ for δ^13^C (min = − 20.0‰, max = − 18.5‰, n = 5). Taking into account the local domestic animals, humans have an enrichment of 3.7‰ for nitrogen and 0.8‰ for carbon. These offsets indicate a varied mixed terrestrial diet, which includes both plant—mainly C_3_, and animal resources, with the exception of BD1092, whose δ^13^C value (− 18.5‰) suggests a mixed C_3_–C_4_ plants consumption. It is likely that meat and dairy products originate principally from sheep and goat, the most represented at the site^65^. Nevertheless, the contribution of other domestic species, like cattle and pigs, should not be underestimated. The similar isotopic results of these species and caprines makes it difficult to identify which species were predominantly consumed by the humans.

The second group includes BD876, BD1132, BD882 and BD794. The median values are 8.7‰ for δ^15^N (min = 8.5‰, max = 9.1‰, n = 4) and − 15.6‰ (min = − 16.5‰, max = − 15.0 ‰, n = 4) for δ^13^C. For both isotopes, significant offsets are evident between the humans and the domesticated animals (∆^15^N_h-f_ = 5.2‰ and ∆^13^C_h-f_ = 5.0‰). The relatively high δ^15^N values suggest a substantial animal protein intake and aquatic foodstuffs could have been part of the diet even though fish remains have not been found in the site. The δ^13^C values, enriched in ^13^C, support an important C_4_ plant consumption. Considering the carbon and nitrogen ratios, marine foodstuffs could have been part of the diet as well, but the altitude and the geographical position of the site make this option quite improbable. This evidence confirms that millets played an essential role in the diet, showing important differences in food choices between the two groups. The first archaeobotanical evidence for millets in Liguria comes from the MBA (Bric Tana)^75^, however the most consistent finds are dated to the FBA-early IA (Monte Trabocchetto)^17^. Consequently, considering the radiocarbon dates of the second group^65^, the δ^13^C values enriched in ^13^C support an important consumption of millets from the FBA-IA transition, consistent with the dates of the archeobotanical results.

The presence of two groups with different diets at Buco del Diavolo is not dependent on sex or age of the individuals because both groups consist of men and women of different age. Therefore, the first explanation to justify this variability could be related to social reasons. Food choices can be a sign of individuals’ differential access to resources, making these choices indirect evidence for the stratification of the community. Lightfoot et al.^76^ highlighted the consumption of millets for some individuals during the Bronze Age (Nadin-Gradina, Croatia) and proposed millet as a low status foodstuff after considering the relationship between δ^13^C and burial type. At Buco del Diavolo, some bracelets and a bronze *torque* are indicative of people of high social status. Unfortunately, as skeletal remains were not articulated, it was not possible to associate the grave goods to the individuals, but it is possible that such differences may also be reflected in diet.

The second explanation could be related to cultural reasons. A viable alternative is that the two groups had different origins and in accordance different dietary habits. Millet was already consumed since the MBA in northern Italy ​​(Verona area)^59^. Therefore, it is likely that some individuals from this area had moved to Liguria. Some archaeological elements typical of the southwestern alpine region are present in the Po basin, in the Provencal areas, in Switzerland, Germany and in southeastern France (RSFO Group of the Urnfield Culture), as attested by bronze grave goods^77^. Thus, the growing exchanges led to an increasing mobility, which could be reflected in different diets.

A third explanation could be associated to the different chronology. The first group is dated to the FBA, while the second group to the beginning of the IA^65^. It is therefore possible that the diets could be the result of different agricultural practices during the two periods: cultivation of C_3_ plants during the FBA and more C_4_ plants during the IA. Moreover, a rapid climate change between 1550–550 cal. BC across the Northern Hemisphere has been recorded^78^ and the paleoclimatic fluctuations responsible for the increase in drier conditions could have led to the diffusion of new crops, like millets. In addition, the spread of human activity affected the ecosystems leading to a decline in woodland cover, the expansion of shrubland and grassland and an increase in soil erosion. There is also evidence for burning from the Late Bronze Age onwards^79^. At present, none of these hypotheses can be excluded, further research on additional isotopes, such as Sr to study the mobility, should provide a clearer picture.

### Ostiglia La Vallona (Lombardia)

The δ^15^N human median is 10.7‰ (min = 10.1‰, max = 13.3‰, n = 5) and the δ^13^C median is − 13.0‰ (min = − 13.3‰, max = − 12.6‰, n = 5). The offsets between humans and animals are 4.4‰ for nitrogen and 8.0‰ for carbon. These results indicate an important consumption of enriched ^15^N and ^13^C resources. The high carbon ratios suggest C_4_ plants consumption like millets, as confirmed by coeval and nearby communities (Olmo di Nogara, Bovolone)^25^, whereas the enrichment in ^15^N could highlight an important animal proteins intake as well as aquatic foodstuffs. Indeed, local communities have exploited the aquatic environment since the EBA-MBA transition, as confirmed by the freshwater animals recovered in the pile-dwelling and Terramare settlements^57^. The isotopic values of freshwater fish from Fondo Paviani^25^ are further evidence of the possible contribution of these resources to the human diet.

Moreover, plants enriched in ^15^N, like the modern cattail (*Typha latifolia)*, a species typical of marshy environments (δ^15^N $\overline{{\text{x}}}$ = 7.0‰, n = 79)^80^ could have been part of the diet, and its consumption is attested since the Mesolithic^81^. Off-site palynological studies in the lower Veneto plain and in Lombardy show a higher rate of afforestation (30–50%), with extensive presence of wooded or marshy areas in the Bronze Age^81–84^. Consequently, wetlands with their typical fauna and vegetation, like bulrush^12^, were potentially exploited for food resources.

According to the archaeological fish isotopic data from the Mediterranean^68,69^, marine resources could have been consumed as well. If marine foodstuffs were consumed, this could lead to the enrichment of both human δ^15^N and δ^13^C values, consequently limiting the importance of C_4_ plants in the diet. However, when considering the local archeozoological evidence and the geographical position of the site, a freshwater food intake is more likely alongside an important consumption of C_4_ plants.

Notwithstanding the limited number of individuals, no dietary differences were detected according to age groups. The only exception is OLV371sub, who shows an enrichment in ^15^N compared to rest of the group but the young age of this individual (1–1.5 years old) suggests that this could be associated to breastfeeding. The only individual whose sex has been estimated is OLV183, determined as male. This individual has the lowest δ^13^C and δ^15^N values. Given the lack of sex data for the other individuals, it is not possible to compare diet between the two sexes.

### Grotta Vittorio Vecchi (Lazio)

The δ^15^N human median is 6.4‰ (min = 5.7‰, max = 6.7‰, n = 4), and the δ^13^C median is − 19.6‰ (min = − 20.1‰, max = − 19.2‰, n = 4). Concerning the ∆^15^N_h-f_, an enrichment of 1.8‰ is recorded. This value is lower than expected, suggesting that the animal protein intake was rather limited. In addition to this, even though the assemblage is based on a low number of records, it seems that legume consumption was likely significant. This hypothesis would be supported by the botanical findings. *Vicia faba* seeds represent 57.3% of the total seeds (2182/3811)^85^. Notwithstanding, it is difficult to assess whether the legume intake was direct or indirect, this because some herbivores show ​​low nitrogen values too and they were likely fed with legumes.

Concerning the ∆^13^C_h-f_, an enrichment of 0.9‰ is recorded. This result suggests that animal proteins were part of the diet. However, the small offset recorded for nitrogen and the likely important legume intake highlight that meat and dairy products were probably not a significant component of the diet.

The carbon and nitrogen data indicate that the individuals we analysed show a homogeneous terrestrial diet as the δ^13^C and δ^15^N are within a narrow range. The plant component was significant and mainly represented by cereals and legumes, as archaeobotanical studies confirm. Based on the available data, the δ^13^C and δ^15^N values ​​do not seem to support the consumption of aquatic resources. Further isotopic investigations conducted on coeval individuals of this area will be necessary to confirm this main dietary pattern where a low animal protein intake and a likely important legume consumption is highlighted. Interesting, some new data from the area seem to confirm it^86^.

A comparison of the adults with the only adolescent shows the latter had the lowest nitrogen value (5.7‰) and the maximum carbon value (− 19.2 ‰). It is likely that this individual had a smaller animal protein intake than the others, but since the contribution of legumes to this group’s diet is important, the picture could be much more complex.

### Trinitapoli (Apulia)

The data obtained by the present study are considered together with the seven individuals analysed by Arena et al.^63^. The δ^15^N human median is 8.3‰ and δ^13^C median is − 19.5‰. The δ^15^N results range between 6.5‰ and 10‰ (n = 14) and δ^13^C values ​​between − 20.0‰ and − 18.7‰ (n = 14). Considering the human and animal offsets, ∆^13^C_h-f_ is 1.0‰ and ∆^15^N_h-f_ is 1.6‰. The carbon enrichment fits into the range between two consequent trophic levels, while the nitrogen one is lower than expected. These results suggest that, even if terrestrial foodstuffs from a C_3_ environment were mainly consumed, animal proteins were not a major source, indicating a diet mainly based on plant proteins, including legumes, as attested by the archaeobotanical records of the area^7^.

Despite the consistency of the results, TRIAB20 and TRIAB22, differ from the main group, showing lower δ^15^N values and highlighting a lower animal protein intake. Tafuri et al.^59^ identified a male with similarly low values at Toppo Daguzzo, suggesting the consumption of similar resources. Several hypotheses can explain these data: (I) a possible common origin for the individuals, explaining analogous dietary patterns; (II) a similar social status—the grave goods associated with the Toppo Daguzzo human indicate this individual was probably a warrior as he was buried with weapons^59^. Regarding the two women of Trinitapoli, it is difficult to associate the grave goods with the individuals because of the collective burials, thus it not possible to define social status except in specific cases^87^. Further investigations to reconstruct their origins could provide additional elements helping to explain these dietary differences.

No significant differences occur between the dietary patterns of the humans from the two sectors of the necropolis, AB and C (δ^15^N, p = 0.805; δ^13^C, p = 0.736). Similarly, no significant differences occur between individuals of different sex (δ^15^N, p = 0.28; δ^13^C, p = 0.97).

### Castiglione (Sicily)

The δ^15^N human median is 7.5‰ (min = 6.3‰, max = 9.3‰, n = 61) and the δ^13^C one is − 19.2‰ (min = − 19.7‰, max = − 18.9‰, n = 61). These values are indicative of a terrestrial diet and, even though no fauna is available for this site nor for the whole island for Later Prehistory, results show that most of the protein intake comes from plants from a C_3_ environment. Nevertheless, humans show a large δ^15^N range suggesting a diversified intake of animal protein. As already highlighted for Trinitapoli, the individuals with the lower nitrogen values could indicate they had a significant legume consumption. The importance of agricultural foodstuffs is supported by studies on caries and other oral observations on the teeth of these individuals^88^. Statistical tests between individuals of different burials show no significant differences for δ^13^C (p = 0.355, H = 6.683) but differences emerged for δ^15^N (p = 0.003, H = 17.756). Significant differences have been detected between the following burials: TB96-TB93 (p = 0.035), TB95-TB93 (p = 0.046), TB119-TB96 (p = 0.041) and TB119-TB95 (p = 0.028) (p-values corrected by FDR). It is likely that the tombs reflect social organization, highlighted by a differential access to resources. For instance, the presence of a bossed bone plaque (*ossi a globuli*), interpreted as a prestigious object, in TB93, could imply that the individuals in this burial were of higher social status than those in the other tombs. Another hypothesis suggests a different origin for some of the individuals. Archaeological data support contacts between Sicily and the Aegean area^89,90^. Moreover, the general heterogeneity of the metric traits and, at the same time, similarities of some of these traits between Castiglione and groups from Greece and other Mediterranean countries, support migration events^91^, as also highlighted by other of southern Italy sites^92^.

Sex and age were estimated only for some individuals. Nevertheless, these parameters do not seem to have influenced food choice. Due to the small number of assessments, statistical tests are only possible for age categories (adults vs. juveniles) and no significant differences occur (δ^15^N p = 0.569; δ^13^C p = 0.56), advising that age did not affect food habits.

## Discussion

The present Bronze Age Italian dataset includes 25 sites, which have provided 355 human and 148 terrestrial and aquatic animal samples. The diachronic overview is presented below, while the geographical comparison, only partially presented in other papers^61,63^, is described in detail in Supplementary Text A.3, Table A.5–A.7, Fig. A.1–A.3.

The individuals of different ages and both sexes are from diverse funerary sites (caves, cemeteries, dwellings) and burial types (collective and individual). Domestic and wild animals establish a dietary baseline for the humans. However, in order to ensure consistency, the offsets between human and animal medians have been based only on the domestic herbivores and omnivors (Supplementary Table A.8). All the published data from Bronze Age sites included in this study are summarized in Supplementary Tables A.2 and A.3.

### The Early Bronze Age

**Figure 2:**
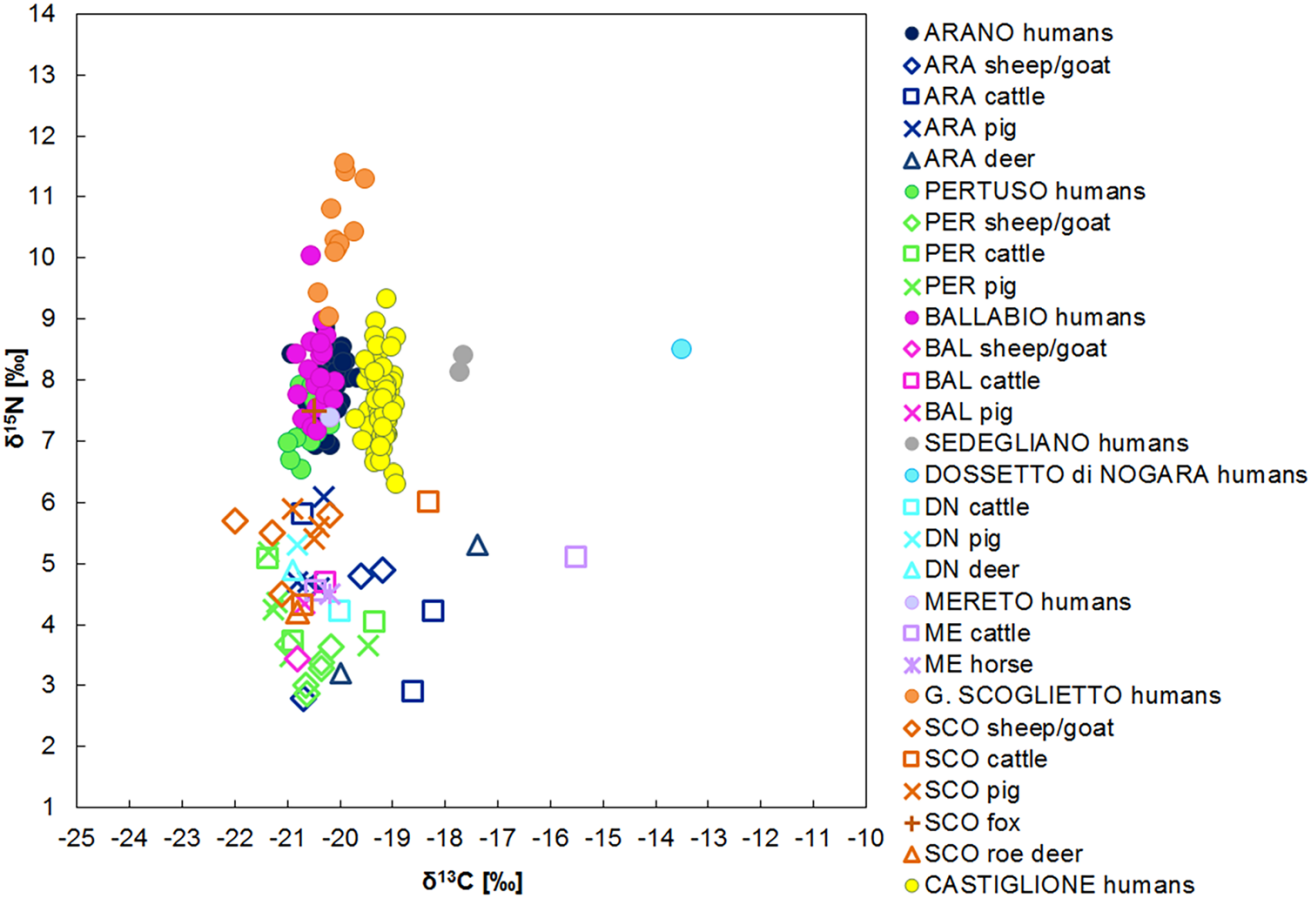
The δ^15^N and δ^13^C human and animal values for the Italian Early Bronze Age sites.

The Early Bronze Age sites, Arano, Ballabio, Dossetto, Mereto, Pertuso, Sedegliano, Gr. Scoglietto and Castiglione, have provided a total of 165 human and 47 animal samples, animal data being provided by six sites (Fig. 2).

Before comparing the humans, the domestic and wild herbivores and omnivores of each site were statistically compared. Significant differences occur among these groups (Supplementary Table A.5), likely linked to the geography and the landscape where the cemeteries are situated. Nevertheless, the animals mainly present a C_3_ terrestrial diet with only small variations, since most of the animal ranges of the sites overlap. The nitrogen values span from 2.8 to 6.1‰ (excluding the fox from Gr. Scoglietto), indicative of the consumption of mostly unmanured resources^93^. The δ^13^C values are widespread, but the 89% of them (41/46) are included in a rather narrow range, spanning from − 22 to − 19.2‰. The five outliers, four cattle (two from Arano, one from Mereto and one from Gr. Scoglietto) and one deer (from Arano) have high δ^13^C values, indicating a C_4_ plant contribution. Concerning Gr. Scoglietto, this evidence is in line with human dental calculus studies, where the presence of millet starch grains and phytoliths have been recorded^94^. The recovered phytoliths may be attributed to different cultivated crops like millets, but also to barnyard grass. In the latter case, grazers like cattle could have eaten C_4_ plants in an anthropogenic environment. For Arano, the fact that a wild animal shows values enriched in ^13^C suggests that C_4_ non-cultivated plants where present in the local environment and wild animals could easily access them.

To minimize the environmental variability, we have compared the offset between the human and domestic animal medians to evaluate differences in the food choices (Supplementary Table A.8) and some patterns are evident. Excluding Dossetto and Sedegliano for δ^13^C values and Gr. Scoglietto for δ^15^N values for which greater offsets are recorded, most of the human groups show a homogeneous mixed terrestrial diet based on animal and C_3_ plant proteins. In fact, the Δ^15^N_h-f_ spans from 2.9 to 3.7‰ and the Δ^13^C_h-f_ from 0.0 and 0.7‰, ranges that fit within the known offsets between two consecutive trophic levels.

A more detailed analysis reveals a uniform distribution of the δ^15^N data for Pertuso, Ballabio, Arano, Mereto, Castiglione and Sedegliano. All the sites show a quite wide range, spanning from 6.3 and 10‰, likely due to a diverse meat and/or dairy products consumption within the communities. The only site which significantly differs from the others is Gr. Scoglietto, where aquatic resources intake has already been suggested^26^. The location of Gr. Scoglietto in a coastal zone and in a lagoon-like area may have led the population to exploit the aquatic environment at the expense of agricultural products. Furthermore, the dental calculus results support the absence of any leguminous species^94^. The apparent absence of these plants in the diet associated to an important intake of animal foodstuffs can explain the enrichment in ^15^N.

In terms of the carbon results, most sites (Arano, Pertuso, Ballabio, Mereto and Gr. Scoglietto) show δ^13^C values falling within a relatively narrow range, from − 21.0 to − 19.7‰. This distribution suggests a diet based on C_3_ terrestrial resources, such as einkorn, emmer and bread wheat and barley associated to wild species differently available according to the regions (e.g., *Quercus sp, Fucus carica, Juglans regia*). Sedegliano, Dossetto and Castiglione show higher δ^13^C values compared to the previous ones. If for Castiglione the slightly higher δ^13^C values could be related to its southern geographical position and to a drier environment^95^, regarding Sedegliano and Dossetto, the higher enriched δ^13^C values are probably due to millets intake. These are likely the first isotopic evidence for millet consumption in Italy and this result is essential to trace the introduction of C_4_ plants to the human diet.

### The Middle Bronze Age

**Figure 3:**
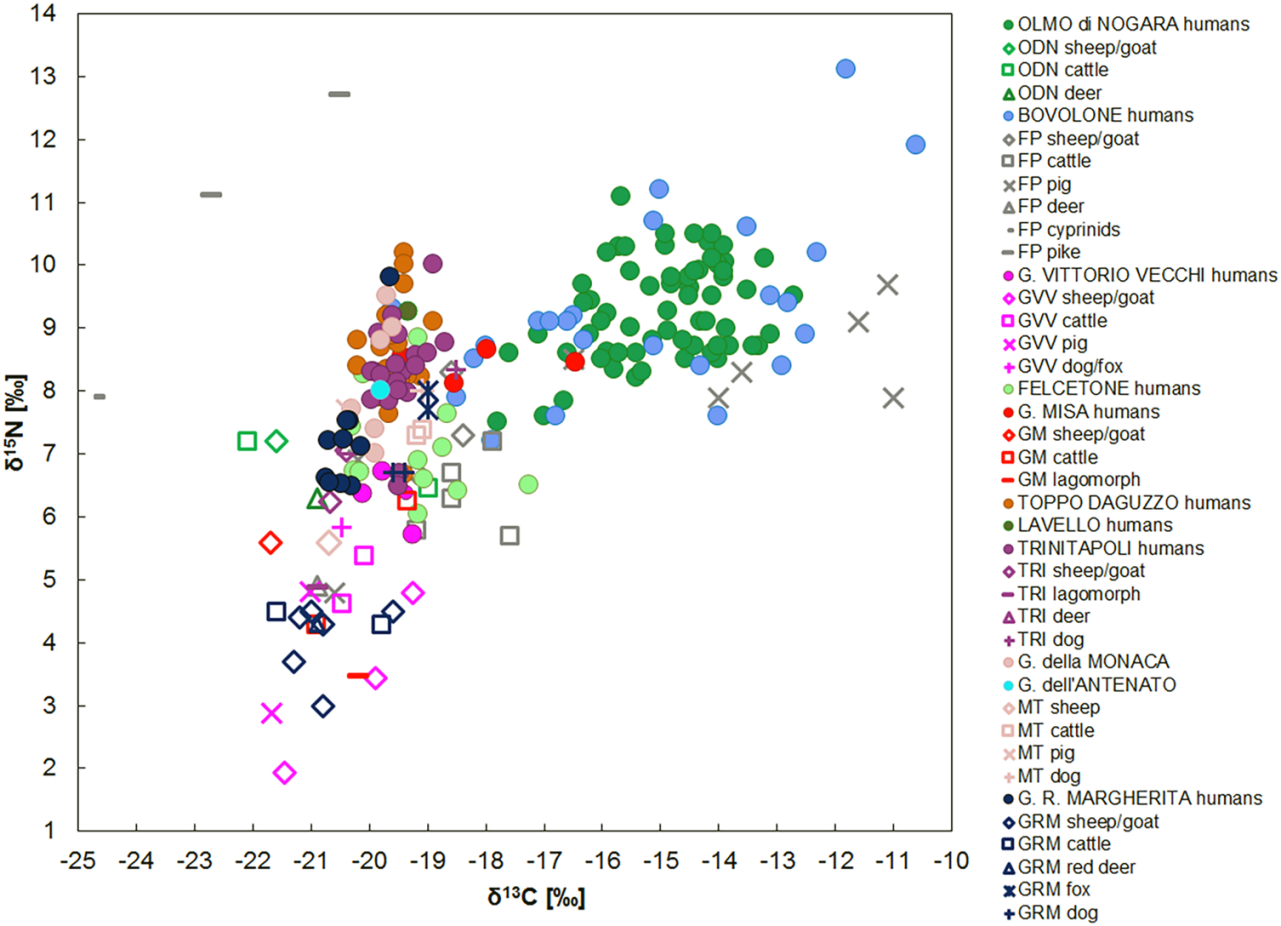
The δ^15^N and δ^13^C human and animal values of the Italian Middle Bronze Age sites.

The Middle Bronze Age includes 13 sites: Olmo di Nogara, Bovolone, Fondo Paviani, Gr. Misa, Felcetone, Gr. Vittorio Vecchi, Gr. Regina Margherita, Lavello, Toppo Daguzzo, Trinitapoli, Gr. della Monaca, Gr. dell’Antenato and Murgia Timone, for a total of 157 humans and 61 animals. This period seems to be a phase of change according to the varied results for all the humans and animals (Fig. 3).

Both carbon and nitrogen animal group data are widespread and statistical differences occur (Supplementary Table A.5). The animals from northern Italy (Olmo di Nogara and Fondo Paviani) show higher δ^15^N and δ^13^C values compared with the sites from the center and the south (Fig. 3). In addition to the environmental and ecological differences which affect the values from the bottom of the trophic chain, the ^15^N enrichment in the northern animals can be interpreted as them grazing close to lands with manured crops or being fed manured plants. The carbon data fit within the typical range of values from a C_3_ environment, with the exceptions of Fondo Paviani, that show enriched δ^13^C values suggesting a C_4_ plants intake, being likely fed food waste^25^.

Given the variability in the animal results, the human diets are compared using the offsets between the humans and their associated fauna. The Δ^15^N_h-f_ span from 0.6‰ at Gr. dell’Antenato to 2.9‰ at Gr. Misa, below what is normally estimated between two trophic levels. As for the northern Italy, Olmo di Nogara and Bovolone, the Δ^15^N_h-f_ are 2.0‰ and 2.4‰ respectively, indicating that the animal protein intake was not very important in this area. The lowest offsets are found in the center and the south of the peninsula, suggesting a limited consumption of animal foodstuffs and supporting a diet mainly based on cereals and legumes. The consumption of legumes seems to have been considerable in central Italy during the Bronze Age and the variety of species is greater than in the Neolithic^16^, as attested by the remnants of a soup of barley, oat, einkorn/emmer wheat and broad beans found at Gorgo del Ciliegio^96^. Therefore, if during the EBA the site of Scoglietto does not show evidence for legume consumption neither from an archaeobotanical nor an isotopic perspective, the δ^15^N values from Felcetone, Gr. Vittorio Vecchi and Gr. Misa confirm the consumption of pulses from the MBA. Additional data from La Pastena cave support the importance of pulses^86^. Based on these results, the MBA represents an important moment of change in terms of the adoption of new foodstuffs for central Italy. For the south of the peninsula, archaeobotanical research supports an increase in the pulse varieties, although broad beans still continue to represent the main taxon^7^. Consequently, according to the nitrogen data for the MBA sites of the entire peninsula, animal protein was not highly consumed during this period, revealing an economy more oriented towards vegetal products rather than animal products.

Regarding the δ^13^C data, interesting patterns appear. The δ^13^C human values are widespread, from − 20.7 to − 10.6‰. A sligh variability has already been identified at the end of the EBA, but at the MBA this pheonomenon is accentatued. Three main groups with specific dietary patterns can be identified. The first includes sites from central and southern Italy: Trinitapoli, Lavello, Gr. dell’Antenato, Gr. della Monaca, Toppo Daguzzo, Gr. Regina Margherita and Gr. Vittorio Vecchi. Here, the Δ^13^C_h-f_ are between 0.1 and 1.0‰, the characteristic enrichment between two trophic levels. Furthermore, the δ^13^C values of these humans overlap and fall within a limited range, from − 20.7 to − 18.7‰, suggesting C_3_ plant consumption. The second group is composed by Gr. Misa and Felcetone. These two communities have common characteristics. First, both sites show wide Δ_h-f_^13^C offsets, (GM = 2.7‰ and FEL = 1.8‰) and, second, they show a wide intragroup δ^13^C range (GM = 2.9‰; FEL = 3‰). The large spread of δ^13^C values, associated with their intermediate position (− 20.3‰ < δ^13^C < − 16.5‰), suggests a mixed C_3_–C_4_ plant intake for both sites. The third group is represented by Olmo di Nogara and Bovolone. Olmo di Nogara’s Δ_h-f_^13^C offset is 5.5‰ and Bovolone’s is 3.5‰. These two groups also show the greatest intragroup variability: the Olmo di Nogara values span from − 17.8 to − 12.8‰ and the Bovolone ones from − 18.5 to − 10.6‰. Thus, millet represented a significant component of the diet of all the individuals. Furthermore, the important Δ_h-f_^13^C enrichment implies that these crops were mostly ingested directly.

According to these data, different trends are evident for the MBA. Some of them are present in the whole peninsula and others are only regional. A general low animal protein intake is detected in all the human groups of this period. In fact, most of the sites do not reach the Δ^15^N_h-f_ offset of 2‰. The greatest inter- and intragroup dietary differences are recorded in the north. Here, diet is mainly based on C_4_ plants (Olmo di Nogara and Bovolone), with evidence for millet consumption also seen among the animals (Fondo Paviani). In central Italy, C_4_ plants started to play an important role in the diet from this period. Nevertheless, C_3_ plants remained the staple food (Felcetone, Gr. Misa, Gr. Regina Margherita and Gr. Vittorio Vecchi) with only some individuals apparently consuming C_4_ plants (Felcetone and Gr. Misa). Furthermore, it seems that pulses were an important part of the diet in this area (Gr. Vittorio Vecchi, Felcetone), as recently supported by other studies as well^86^ In the south, there is a lower intra- and intergroup dietary variability and C_4_ plants were not routinely consumed (Toppo Daguzzo, Lavello, Trinitapoli, Gr. Della Monaca and Gr. dell’Antenato). It is, therefore, probable that millets started to be routinely consumed from the north to the south of the peninsula, with a likely gradual replacement of the more common wheat and barley.

### The Recent and Final Bronze Age

There are four Recent and Final Bronze Age funerary sites: Ostiglia, Buco del Diavolo, P. di Zambrone and Gradisca di Codroipo, for a total of 16 humans and 40 animals. These later Bronze Age phases are not well-represented because of the limited number of funerary sites with inhumations, following the introduction of cremation in most of Italy.

**Figure 4:**
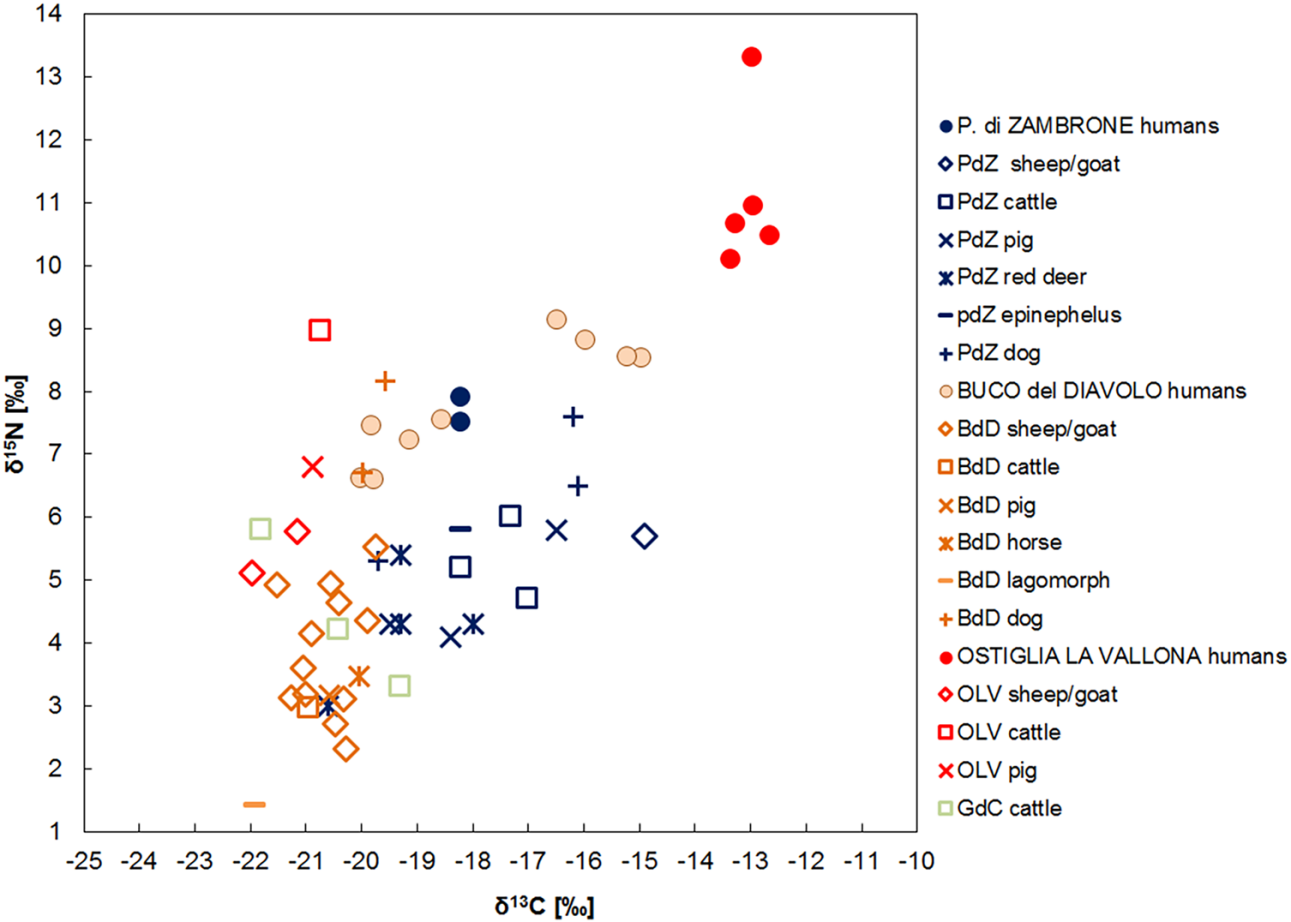
The δ^15^N and δ^13^C human and animal values for the Italian Recent and Final Bronze Age sites.

The animal nitrogen and carbon values are widespread and significant differences occur among the groups (Supplementary Table A.5). This wide variability is particularly evident for carbon values because the animals from P. di Zambrone, at the south of the Peninsula, are enriched in ^13^C compared to the ones from the north. Even though differences linked to the diverse environmental conditions could not be excluded because of the different latitudes, the results are more likely related to different diets. The specimens from the north had a diet mainly based on C_3_ plants, whereas the ones from the south, at least some of them, grazed upon seaweeds or, more likely, had a mixed C_3_–C_4_ plants consumption, in accordance with the archaeobotanical remains of P. di Zambrone^97^ (Fig. 4).

According to the human and animal offsets, different human dietary habits are evident. If the Δ^15^N_h-f_ and Δ^13^C_h-f_ at P. di Zambrone are not remarkable, suggesting that both humans and animals had a C_3_–C_4_ mixed terrestrial diet, the ones from Ostiglia and Buco del Diavolo are considerable, 8.0‰ and 2.1‰ respectively, indicating a human inter- and intragroup food variability and the significant consumption of ^13^C enriched resources. As mentioned, millets started to be consumed from the MBA and these results demonstrate that this trend continues later. Furthermore, humans mainly consumed C_4_ plants directly, since animals do not show any C_4_ signal. At Ostiglia and Buco del Diavolo, the Δ^15^N_h-f_ are 4.4‰ and 4.0‰ respectively, suggesting that animal protein and/or aquatic resources had an important role in the diet. Taking into account the geographical area of the two sites, Buco del Diavolo in Liguria, over 1000 m. a.s.l., and Ostiglia, in the Po Basin, in the center of one of the most economically developed areas during the Bronze Age, it is not surprising that such a wide variety of resources was consumed. In addition, studies on animal management during the FBA in the Po Basin show that even if the subsistence economy was based on agricultural activities, pigs were slaughtered before reaching adulthood to exploit the higher quality of meat and cattle and caprines were reared until adulthood to exploit their secondary resources^42,98^. These results, which are consistent with animal protein intake, indicate that specific attention was paid to animals depending on their resources and their different purposes.

The great diversity in the dietary practices for the RBA-FBA compared to the previous phases is present at the intra-group level as well, particularly at Buco del Diavolo, as previously described. This is the first time that such different dietary strategies are recorded between individuals from the same community in the Italian Peninsula.

### Insight into the changes in Bronze Age dietary patterns: an integrated approach

Stable isotope data highlight drastic changes in food habits during the Bronze Age. The new results combined with published data have revealed the emergence of three phenomena: (I) the introduction of C_4_ plants in the diet; (II) the increasing importance of legumes; (III) the increased consumption of animal products.

**Figure 5:**
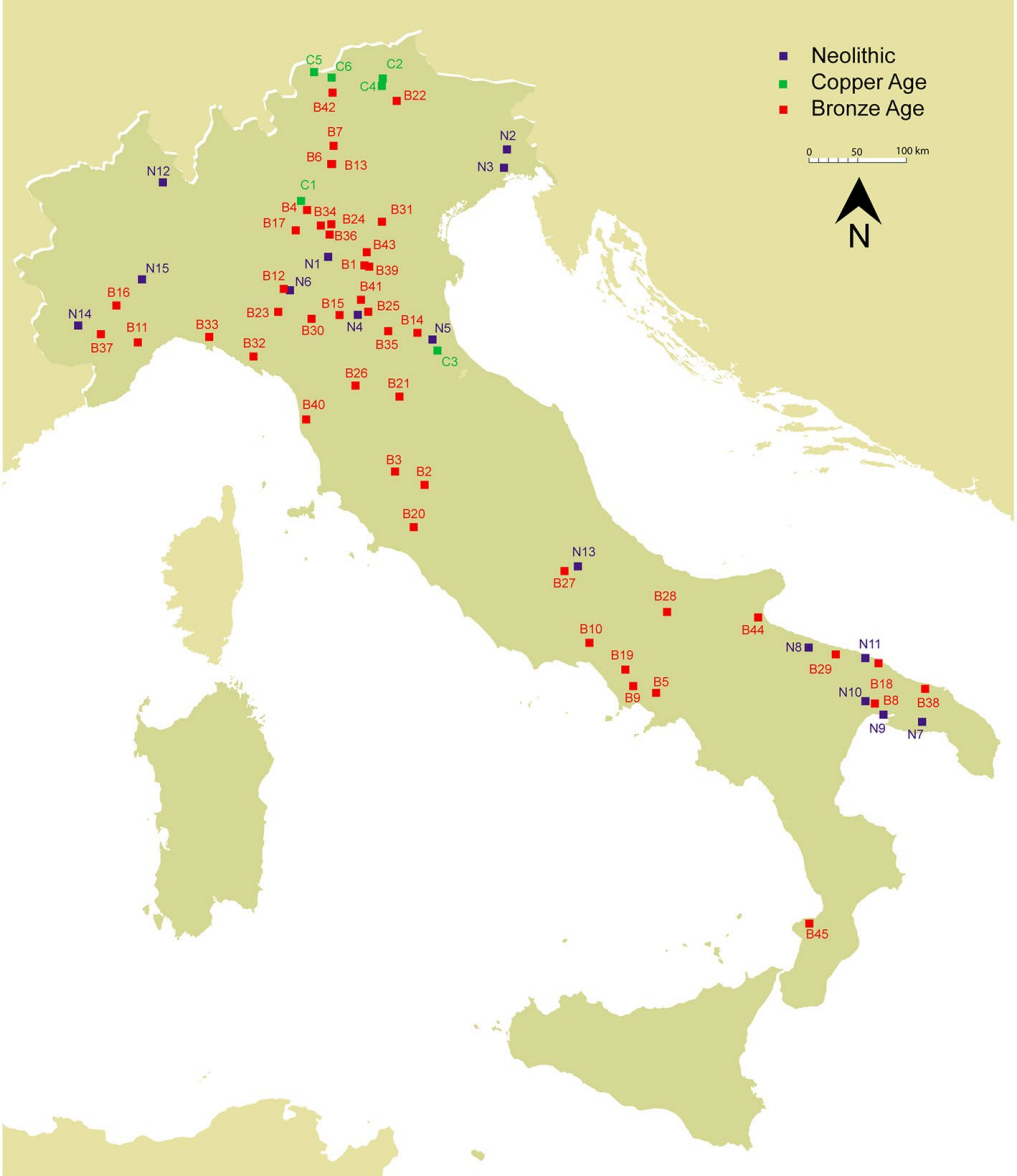
Map of Neolithic, Eneolithic and Bronze Age sites where archeobotanical remains of common and foxtail millet have been found (the list of the sites and the relative references are in Supplementary Information 2, Table B).

The consumption of C_4_ plants represents the first major change in terms of new crops since the introduction of agriculture, and it has also been identified as an example of “food globalization”^21^. In order to trace the origins of this new practice, botanical research together with isotopic data from all of Italy have been evaluated. Some of the archaeobotanical evidence supports the spread of millets starting in the Neolithic and throughout the Bronze Age. First, there is a significant increase in the number of sites where millet can be found (Fig. 5 and Supplementary Information 2, Table B). Second, the distribution area is wider, including sites in southern Italy. Third, the findings are varied, representing both seeds and chaff. The greatest amount of millets has been found in the Po Basin, highlighting how crucial this zone was for the spread of millet throughout the peninsula (Fig. 5 and Supplementary Fig. A.4).

**Figure 6:**
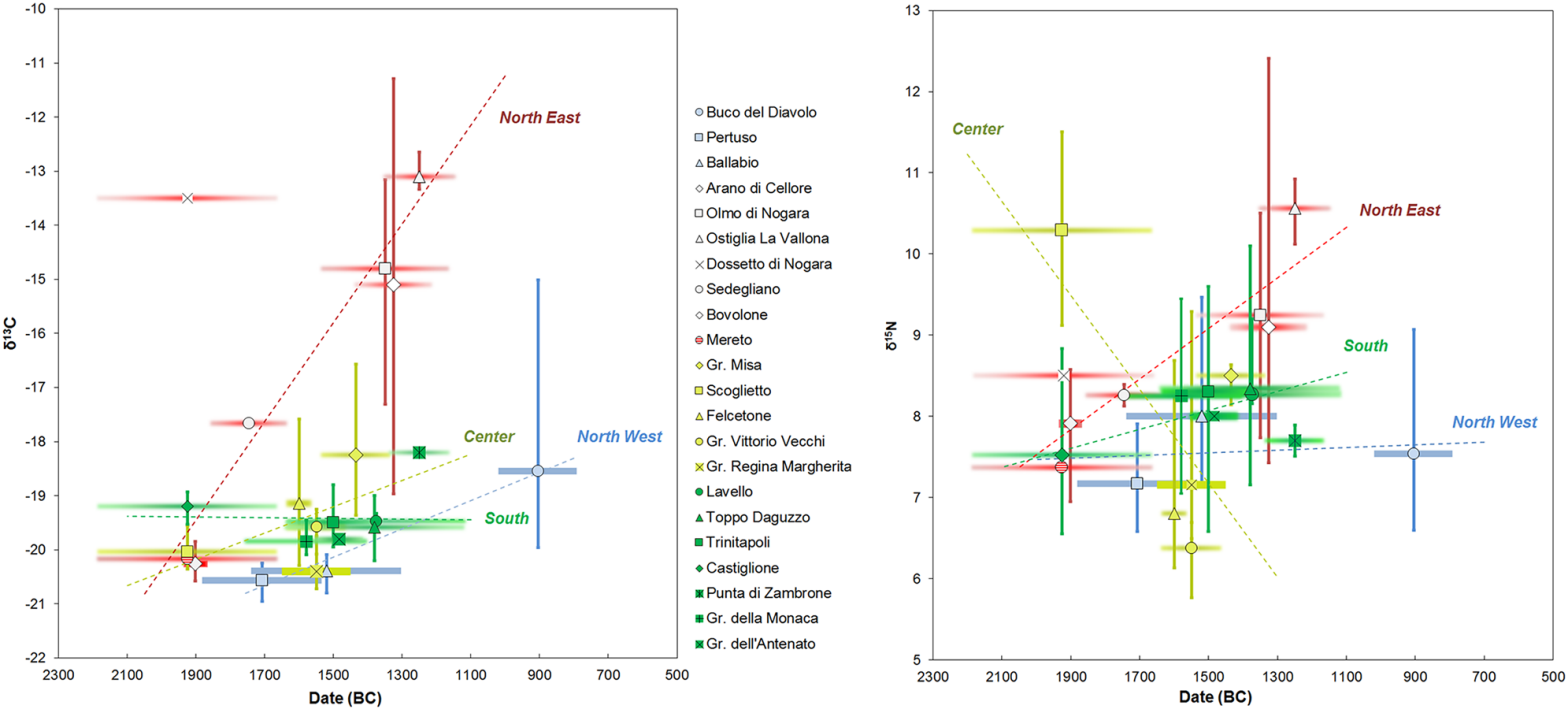
Distribution of δ^13^C (left) and δ^15^N (right) human group medians according to their chronology. Horizontal bars with blurred strokes indicate the absence of absolute dates. Horizontal well-defined lines indicate chronological periods determined through radiocarbon dates. Regression lines are calculated and weighted according to the number of individuals for each site: n = 1, w = 0.25, n = 2–3, w = 0.5, n > 3, w = 1.

**Figure 7:**
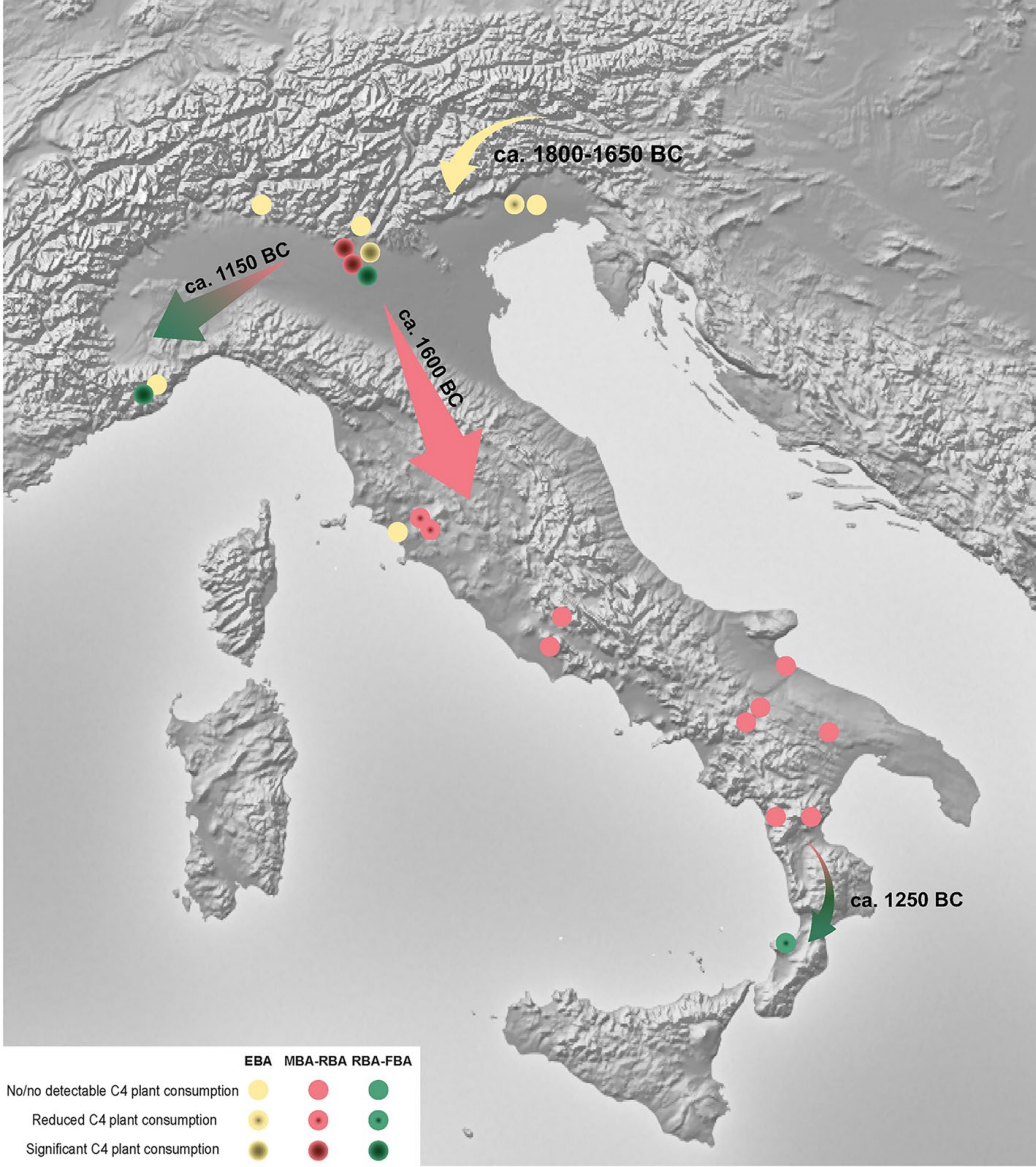
The “Italian millet road”. Map of Bronze Age Italian sites for which isotopic analyses have been carried out. The color intensities indicate differential consumption of C_4_ plants according to chronological period (The map is elaborated using “Natural Earth. Free vector and raster map data @naturalearthdata.com” available at https://www.naturalearthdata.com/downloads/10m-raster-data).

Isotopic studies in Italy show no evidence of C_4_ plant consumption for the Neolithic^65,71,72,98–105^, even if doubtful consumption seems to have been recorded in some individuals at Mora Cavorso^106^. Neither were millets commonly consumed during the Eneolithic^106–110^. Even if an enrichment in ^13^C could be induced by several causes, i.e. marine foodstuffs, from this study it is evident that increasing higher δ^13^C values are mainly due to C_4_ plants. The first evidence for C_4_ plants intake is detected in the EBA and EBA-MBA in the northeast of the peninsula—Dossetto and Sedegliano. All other evidence dates to the MBA in northern Italy—Olmo di Nogara and Bovolone and to a lesser extent in the central regions—Gr. Misa and Felcetone. The C_4_ signal is well established later on in the Bronze Age, the RBA-FBA, mainly in the north—Ostiglia, Buco del Diavolo, with some evidence for the south at P. di Zambrone (Figs. 6 and 7).

When combining isotopic and archaeobotanical data, a more detailed scenario emerges for the identification of the “Italian millet road”. Where botanical studies infer the presence/absence of C_4_ plant species, stable isotopes analyses quantify the direct or indirect intake of these plants. In fact, archaeobotanical remains reveal the spread of millets from northern Italy since the Neolithic (Fig. 5), but the isotopic data indicates humans only started to regularly consume millets from the EBA-MBA transition, beginning in the northeast, then the center and finally the south of the peninsula (Fig. 7). Radiocarbon dates performed on charred broomcorn millet grains confirm this trend. Filipović et al.^21^ estimate the start of millet cultivation in the Po Basin between 1570 and 1410 cal. BC (95.4% probability) at the beginning of the full development of the Terramare culture^111^. Furthermore, biomolecular analysis performed on on-site archaeological sediments (i.e. Fondo Paviani) confirms the presence of broomcorn millet by the biomarker miliacin^112^. Millets were probably consumed in previous phases, like the dental calculus from Gr. Scoglietto suggests^94^, but in such a small quantity that it cannot be isotopically detected.

In southern Europe, the first evidence for millet consumption comes from Greece at Perachora^113^ in the Early Helladic (3100–2100/2050 BC) and at Rymnio and Spathes^114^, Pineiada, Aghia Triada and Almyri^114–117^ dated to the Late Helladic (1700/1675–1075/1050 BC). Croatia, Spain and France, as well as countries beyond the Alps, like Switzerland, Germany and Austria, show sporadic C_4_ signals, but only from the Late Bronze Age onwards. Conversely, during the IA, results are more homogeneous exhibiting the typical values of millet consumers^76,117–127^.

It seems that the Caucasus area represents a key zone for the spread of millets into Europe because millet intake is recorded from the first half of the 2nd millennium BC^128,129^. The identification of an ‘Isotopic Millet Road’ from China to Central Europe and the Mediterranean reveals that C_4_ dietary signatures reflect early links, in the form of migration and/or resource transfer, between the Bronze Age inhabitants of China and Europe^130^. These results show that the westward expansion of millet as a foodstuff into Europe was a rather fast and global phenomenon instead of a gradual one. This picture is confirmed by Filipović et al.^21^, whose research indicates the earliest occurrence of millet at the middle of the 2nd millennium BC, supporting its spread “at only slightly different times in different parts of Europe”. Their Bayesian model shows that it was cultivated in southeastern Europe (Ukraine) at the earliest during the sixteenth century BC, promptly spreading during the fifteenth-fourteenth centuries BC to most of southeast and Central Europe and only in the late 13th/early twelfth centuries BC into northwest Europe. Consequently, the radiocarbon-dated caryopses indicate that millet did not arrive in Europe during the Neolithic and that most of the Neolithic attributions should therefore be doubted. Furthermore, considering regional variability, the first millet consumption in Europe, including Italy, and the earliest radiocarbon dates on broomcorn millets seems to overlap. This evidence suggests that the adoption of millet as part of a human diet was heterogeneous and was contingent on the area and the site.

Why are new crops like millets introduced? An important demographic growth associated to an increasing socio-political and economic connectivity between the Mediterranean area and continental Europe may provide the answers. This connectivity has been crucial not only for the exchange of ideas, lifestyle and technologies but also for objects, products (crops and animals) and resource transfer (e.g., raw materials, prestigious goods), as demonstrated by archaeological evidence and confirmed by bioarchaeological and genetic data^130–133^. Contacts between the Carpathian basin and northern Italy, through Austria and Slovenia, have been well identified, particularly from the Bronze Age^134^, and millet was probably part of this “package exchange”. Millets were likely introduced to diversify the agricultural production and to ensure harvests when annual winter wheat and barley crops were not sufficient.

Pivotal agricultural system changes have been recorded since the Eneolithic: these mainly consisted in the transition from a self-sufficient horticultural subsistence strategy to a more extensive agricultural system, where intensive production was managed by a more complex society and when a gradual demographic growth is recorded. The ever increasing areas dedicated to agricultural and pastoral activities caused heavy deforestation with the over-exploitation of land and caused the soil to dry up. This aridity led to the introduction of innovative strategies, including a network of irrigation ditches as well as the cultivation of less demanding plants, such as millets^14,135^.

In addition, episodes of dryness are recorded in the EBA, as different proxies in the Italian sub-alpine lakes show^84,136^, they are also observed as a prominent warm spell in the residual ∆^14^C curve. These short events could have affected variations in crop cultivation. Therefore, the increasing aridity caused by climatic fluctuations associated to heavy deforestation and the over-exploitation of soils^12,14,137^, supports the introduction of new, more tolerant species, less sensitive to stressful conditions.

However, some questions arise: why was millet mainly consumed only in the north of Italy? Why was millet differently consumed within groups (e.g., Buco del Diavolo)? According to the evidence from other European sites, variable intake could be related to social, cultural and geographical factors^118,120–123,127^. Indeed, millet consumption is attributed to lower social status individuals in Croatia and Germany^76,123^. Nevertheless, the C_4_ signal, when detected in the Italian sites, is usually recorded for all the community’s individuals, like at Olmo di Nogara, Bovolone and Ostiglia, even if this last site is just represented by few individuals. It is, therefore, likely that millet was easily accessible in Italy.

The second phenomenon concerns the growing importance of legumes both in diversifying the human diet and for improving the fertility of the soil through crop rotation^9^. Pulse cultivation was mainly based on broad bean all across Italy and, depending on the site, significant quantities of pea, grass pea, red pea, lentil, common vetch and ervil have been found^6^. Indeed, in addition to a greater abundance of legume seeds, there is also a wider variety of species, especially compared to the Neolithic^138^. If this picture is quite clear for the north, it is less evident for the center and the south of Italy. Despite the increasing number of taxa, the presence of legumes is often controversial. Sometimes it is not possible to ascertain whether these plants were weeds or were cultivated for their physiological characteristics^6,7^. Isotopic analyses contribute to discriminating between legumes as part of the diet or not and determining whether they were cultivated or not. Indeed, legume consumption can be detected when associated with a low animal protein intake. This is the case of Gr. Vittorio Vecchi. Furthermore, the archaeobotanical data from this site show that bean seeds represent more than 50% of the total remains^16,139^, revealing the significance of legumes for this community. Consequently, these new data, associated with other coeval sites of the region and additional unpublished data^86,110^, provide evidence for the increasing importance of legume consumption during the Bronze Age for central Italy. This site clearly showcases how multidisciplinary studies that combine isotopic data and archaeobotanical research are essential for the reconstruction of subsistence practices and for defining the significance of these plants in human diet.

Finally, the third evidence is a rise in animal protein consumption. Indeed, a gradual enrichment in ^15^N in the individuals of the northeast, northwest and in the south throughout the Bronze Age is recorded (Fig. 6). The higher nitrogen values could be attributed to different causes, like the consumption aquatic resources as well as the increase in aridity recorded from the EBA onwards cannot be underestimated. However, the global increase of dryness could have negatively affected crop cultivations, explaining the greater interest in meat and secondary products highlighted for the end of the Bronze Age. In northern Italy, pollinic analyses indicate that only 30–40% of the total deforested area was used for agriculture and the rest was for livestock breeding^140,141^. Indeed, RBA Terramare sites, like S. Rosa di Poviglio and Montale, show an increasing number of caprines at the expense of pigs and cattle. Goats prefer open spaces and can tolerate drier pastures. Therefore variations in subsistence practices can be due to readjustments of economic strategies in reply to changes in the ecosystem. Towards the end of the Bronze Age, a worsening of the agricultural yield is recorded at Montale and Rosa^14^. Cereal cultivation significantly declines, while the uncultivated areas spread^142^. It is therefore possible that several years of dryness, in conjunction with economic and social factors, contributed to precipitating a situation that was already beyond the thresholds of sustainability, pushing the population to invest in new resources, like in a pastoral economy for meat and secondary products.

Even though the species exploited as well as the animal size varied according to geographical area^143^, research into livestock farming in Liguria, for instance, supports an increased interest in the pastoral economy. During the Neolithic, during times of important demographic growth, pastoral practices, which included the use of caves as stables and suckering to provide foliar fodder, were introduced^144,73^. From the end of the Neolithic, transhumant pastoralism spread and contributed to the thinning out of coniferous forests in the mountains to obtain, through the practice of controlled fires, new highland pastures^17^. During the Eneolithic and Bronze Age, when the climate deteriorated, the practice of transhumance towards highland pastures was consolidated^145^.

## Conclusions

This research presents the first global overview of human and animal isotopic data used to reconstruct dietary patterns for Bronze Age Italy. As some areas are still isotopically unexplored, the present scenario may change with new studies. However, based on the present data, some trends have been highlighted.

Results suggest that the MBA was a period of major change and that the north of Italy, particularly the northeast, seemed to have been at the forefront of this dynamic, where new trends were introduced that spread into other areas of the peninsula. Changes in dietary patterns are clearly detected, resulting in either global or more regional patterns. Indeed, we highlighted (I) an increased consumption of broomcorn and foxtail millet, mainly for humans from the EBA/MBA transition, (II) a greater legume consumption, particularly for the populations of the center and south of Italy from the MBA onward and (III) a likely increase in meat and/or dairy product intake at the end of the Bronze Age. Several factors could have been responsible for these changes, including the climatic fluctuations that resulted in more arid soils, alongside the strong impact of human activities. Indeed, anthropic activities like deforestation, the over-exploitation of soils for agriculture and livestock breeding and the increase of metallurgic production could have reduced soil fertility. Consequently, the need for a more diverse diet arose, leading to the adoption of more tolerant crops and further resources to exploit. In addition, the increase of trade and the related economical activities with other Mediterranean and continental European areas points to exchanges at different levels, not only in terms of prestigious goods, i.e. ivory, amber and silk, but also in terms of local agricultural practices, ways of life, traditions and, consequently, socio-economic systems.

This study is a first step towards a more in-depth understanding of the Italian dietary patterns during the Bronze Age, which is still a largely unexplored period in terms of biomolecular investigations, and it represents also a new piece of the gigsaw of the global understanding of Bronze Age societal developments. Further isotopic data on botanical, animal and human remains are necessary to improve the resolution of our analyses and to gain a more fine-grained depiction of subsistence strategies during a time of rapid socio-economic change.

## Methods

### Carbon and nitrogen isotope analyses on human and animal bone collagen

Protocols proposed by Longin et al.^146^ and modified by Bocherens^147^ were followed for bone collagen extraction for Pertuso, Buco del Diavolo, Ostiglia La Vallona, Gr. Vittorio Vecchi, Trinitapoli and Castiglione. The collagen extraction was performed at the LAMPEA Biochemistry Unit (Aix-en-Provence, France) and the isotopic ratios were measured at Iso-Analytical Ltd (Europa Scientific 20–20, Crewe, UK). The isotope ratios are reported as delta that has been defined according to IUPAC (International Union of Pure and Applied Chemistry):

$$\delta = \left( {{\text{Rs}}/{\text{Rst}}} \right) - {1} = {1}0^{{3}} [\left( {{\text{Rs}}/{\text{Rst}}} \right) - {1}].$$

where Rs and Rst are the isotope ratios ^15^N/^14^N and ^13^C/^12^C of the isotopic abundances of ^15^N, ^14^N, ^13^C, and ^12^C, and ‰ = 10^–3^. The analytical precision of both samples and standards is 0.1‰ for both δ^15^N ​​and δ^13^C.

As for the published data, the samples from Ballabio, Felcetone, Gr. Misa, Gr. Scoglietto were analyzed following the above cited protocol as part of the same research^26,61^. The sites of Arano, Olmo di Nogara, Mereto, Sedegliano, Gradisca di Codroipo, Lavello, Toppo Daguzzo, Dossetto di Nogara, Bovolone, Fondo Paviani, Gr. Regina Margherita and Punta di Zambrone followed a similar collagen extraction protocol applied to a fragment of cortical bone^25,59,60,62,64^. The samples from Gr. della Monaca, Gr. dell’Antenato, Toppo Daguzzo, Trinitapoli and Murgia Timone, analyzed by Arena et al.^63^ followed a similar protocol but they were also ultrafiltered.

### Analytical method

0.5 g of powdered bone was soaked in HCl (1 M, 20’) for demineralization and in NaOH (0.125 M, 18 h) to eliminate contaminants and finally dissolved in HCl (pH = 2, 17 h, 100 °C). The residue was then frozen (> 5 h, − 60°) and freeze dried for 48 h. Isotopic ratios and elemental contents were provided by Elemental Analyzer coupled to Isotope Ratio Mass Spectrometry at Iso-Analytical Ltd (Europa Scientific 20–20, Crewe, UK).

Isotopic accuracy was monitored through routine analyses calibrated against international standards: bovine liver (IA-R042), a mixture of ammonium sulfate (IA-R045) and beet sugar (IA-R005), and a mixture of sugar cane (IA-R006) and ammonium sulfate (IA-R046). The analytical precision of both samples and standards is 0.1‰ for both C and N. Several preservation criteria have been considered to check for collagen integrity: collagen yields greater than 1%, %C ≥ 30% and %N ≥ 10%, and C/N ratios ranging between 2.9 and 3.6^146–150^.

### Statistical analysis

Statistical analysis used R 3.6.1^151^ and the coin package v. 1.3–1^152^. Considering the small sample size, the non-parametric tests of Kruskal–Wallis (Monte Carlo approximation, 10,000 replicates) and exact Wilcoxon Mann–Whitney are used to assess for significant differences in δ^13^C and δ^15^N. In the case of multiple tests, in order to identify as many significant comparisons as possible while still maintaining a low false positive rate, the False Discovery Rate (FDR) is applied on the results obtained by the Wilcoxon Mann–Whitney tests^153^. Only corrected p-values are presented. A 0.05 probability (p < 0.05) is considered significant.

## Supplementary Information

Supplementary Information 1.

Supplementary Information 2.

## Data Availability

All data needed to evaluate the conclusions in the paper are present in the paper and in the Supplementary Information 1 and 2.
