# Supplementary material for: A multi-proxy bioarchaeological approach reveals new trends in Bronze Age diet in Italy
Source: Sci Rep. 2022 Jul 16;12:12203. doi: 10.1038/s41598-022-15581-0 (PMC9288517; doi:10.1038/s41598-022-15581-0)
Supplement: Supplementary file 1 — Supplementary Information 1. [file 41598_2022_15581_MOESM1_ESM.pdf]

A multi-proxy bioarchaeological approach reveals new trends in Bronze Age diet in Italy

Supplementary information 1

Alessandra Varalli[1,2,3*] (orcid: 0000-0001-5502-9204)

Jacopo Moggi-Cecchi[2] (orcid: 0000-0001-5055-8369)

Gwenaëlle Goude[1] (orcid: 0000-0002-3008-3607)

[1]Aix Marseille Univ, CNRS, Minist Culture, LAMPEA, Aix-en-Provence, France

[2]Department of Biology, Laboratory of Anthropology, University of Florence, Italy

[3]CaSEs Research Group, Department of Humanities, Universitat Pompeu Fabra, Barcelona, Spain.

[*]Corresponding author: alessandra.varalli@upf.edu; alessandravaralli@gmail.com (AV)

Index

Text A.1: Site archaeological context ……………………………………………………………… 3

Text A.2: State of preservation …………………………………………………………………….…. 6

Text A.3: Comparative analysis: the geographical approach …………………………….…………… 6

Figure A.1: The δ^{15}N and δ^{13}C human and animal values of the sites in northern Italy...……........… 8

Figure A.2: The δ^{15}N and δ^{13}C human and animal values of the sites in central Italy …….…......… 9

Figure A.3: The δ^{15}N and δ^{13}C human and animal values of the sites in southern Italy ………….... 11

Table A.1: The sample dataset …………………………..……………………………………………. 13

Table A.2. Summary descriptive statistics for all the specimens available (herbivores, carnivores and omnivores) ……………………….……………………………………………………..……… 13

Table A.3. Summary descriptive statistics for all the human isotopic results. ………….…………… 13

Table A.4: Spearman Rho test for δ^{15}N and δ^{13}C with the carbon and nitrogen elemental composition and C/N ranges. ……………………………………………………………………….…….…... 20

Table A.5: Kruskal Wallis test for animals (excluding fox and dogs). …….…….………….…..….…... 20

Table A.6: Kruskal Wallis test for the humans from central Italy; Exact Wilkoxon-Mann-Whitney test for the humans from central Italy with FRD correction. …….…………….…………..….…... 21

Table A.7: Kruskal Wallis tests for human groups from southern Italy …….…….……………...... 22

Table A.8: Offsets between the domestic animal (sheep/goat, cattle, pig, horse) and human medians for each Bronze Age site. To be consistent between sites, no wild animals have been included because not all sites have them. ………………………………………………………………….………..... 22

Figure A.4: Map of Early, Middle and Recent-Final Bronze Age sites where archeobotanical remains of common and foxtail millet have been found (the list of the sites is in Supplementary Table B) … 23

References ……………………………………………………...…………………….………... 24

Text A.1: Site archaeological context
The description concerns only the sites for which isotope data are presented here for the first time (in bold).

The north of Italy

Site	*Locality, Region*	*Altitude (a.s.l.)*	*Chronology*	*Samples*	*Isotopic study*	*Essential references*
**Pertuso**	**Imperia, Liguria**	**1330 m**	**EBA-MBA (1883-1531 BC)***	**Humans, 14 Animals, 14**	**this study**	**1–3**
**Buco del Diavolo**	**Imperia, Liguria**	**1430 m**	**FBA-EIA (1020-790 BC)***	**Humans, 9 Animals, 18**	**this study**	**1,4–6**
Ballabio	Lecco, Lombardy	700 m	EBA-MBA (1740-1300 BC)*	Humans, 22 Animals, 3	7	8–11
**Ostiglia La Vallona**	**Mantova, Lombardy**	**13 m**	**RBA**	**Humans, 19 Animals, 4**	**this study**	**12–14**
Arano di Cellore	Verona, Veneto	210 m	EBA (1926-1877 BC)*	Humans, 54 Animals, 13	15	16,17
Olmo di Nogara	Verona, Veneto	18 m	MBA-RBA	Humans, 64 Animals, 5	18,19	20
Bovolone	Verona, Veneto	24 m	MBA/RBA	Humans, 24 Animals, 0	19	21
Dossetto di Nogara	Verona, Veneto	20 m	EBA	Humans, 1 Animals, 3	19	22
Fondo Paviani	Verona, Veneto	16 m	MBA/RBA transitional phase-FBA	Humans, 0 Animals, 21	19	23
Mereto	Udine, Friuli Venezia Giulia	98 m	EBA	Humans, 1 Animals, 3	18,19	24,25
Sedegliano	Udine, Friuli Venezia Giulia	70 m	EBA-MBA	Humans, 2 Animals, 0	18	26
Gradisca di Codroipo	Udine, Friuli Venezia Giulia	36 m	RBA-FBA	Humans, 0 Animals, 3	19	27

*refers to ^{14}C dates. For further information see the relevant references.

Pertuso is a natural cave situated in the Argentina Valley, Triora district, in the Liguria hinterland at 1330 m a.s.l. This great cave, dated to the Early-Middle Bronze Age, is composed of two separate chambers and several narrow tunnels. It contained limited archaeological evidence, consisting of some archaeobotanical macroremains and grave goods of different types, such as potsherds, bronze tools and shells ornaments [2]. An archaeobotanical analysis on scattered charcoal recovered from three fireplaces in different stratigraphic levels indicates that the environment was dominated by conifers (e.g. *Pinus diploxylon*, *Pinus* cf. *sylvestris*). The abundance of *Laburnum* sp. (78/177 charcoals) highlights the variability of the species present at that time in the area investigated [2].
Associated with abundant animal remains represented by sheep and goat (O*vis/Capra*), cow (*Bos taurus*) and pig (*Sus domesticus*) [1], human remains dating from the final phase of the Early Bronze Age and the beginning of the Middle Bronze Age were recovered [2,3]. The cemetery contains collective burials and the skeletons were not in articulation. Human osteological analyses led to an estimate of 18 individuals of different ages-at-death and both sexes [28,29].

Buco del Diavolo is a site located at 1430 m asl in the Argentina Valley, Triora district, in Liguria, just some kilometres from Pertuso. It is a natural karstic sinkhole 15 m deep. Since 1998, several excavations have uncovered a large amount of animal and human remains in association with exceptional bronze grave goods. The use of the site as a cemetery spans from the Final Bronze Age to the early Iron Age, with some sporadic remains recovered at the top of the pitch and dated to the Roman period, as radiocarbon dates from some of the human remains show [6]. The archaeological assemblage is varied and includes some little ceramic fragments attributed to common pottery and significant bronze ornaments like a *torque* and eight well preserved decorated

armillas [4,5]. Combined with archaeological remains, isolated fragments of charcoal have been recovered. The distribution of the archaeobotanical remains suggests these fell into the cave from outside rather than the presence of fireplaces within. Furthermore, the bad preservation of the remains did not allow for the identification of the species or for radiocarbon dating [30]. In addition, several animal remains were also found. The most abundant are sheep and goat (O*vis/Capra*)*,* including pregnant individuals, as revealed by the presence of fetal remains [30]. In association with sheep and goats, there were also a considerable amount bones and teeth of *Bos taurus*, *Equus caballus*, *Canis domesticus*, *Lepus* sp. and *Sus domesticus*. Most of the animals were found in anatomical connection and were in a good state of preservation [30]. Conversely, the human remains were not in anatomical connection, with the rare exception of some anatomical areas (e.g., segments of several vertebrae). The funerary data show that these are primary burials. Analyses on the human remains led to the identification ten individuals of different ages-at-death and both sexes [31].

Ostiglia La Vallona is a large necropolis in the Po Valley, between the rivers Adige and Mincio, just five kms north-west from the village of Ostiglia, near Mantua. The site presents a considerable stratigraphic sequence extending from the Mesolithic to the Roman period. The layers attributed to the Bronze Age are specifically dated to the Recent Bronze Age, according to the relative chronology from studies conducted on archaeological remains associated with human remains [12–14]. More than 400 graves have been found since the first archaeological campaign in 1985. The site is a bi-ritual necropolis due to the presence of both inhumations and cremations. This kind of cemetery is common in the west of the Veneto for the period investigated. For this study, the individuals from 20 tombs (16 single and 4 double burials) found during the excavations in 1986 and 1988 were considered. Grave goods such as pins, bronze earrings and needles were rarely associated with the individuals. No weapons or ceramics were recovered. Some animal remains represented by *Bos taurus*, *Sus domesticus* and *Ovis/Capra* were found. The human remains are poorly preserved due to recent agricultural activities in the necropolis. The individuals included in this study are 26, representing different ages-at-death and both sexes, and a high infant mortality rate has been highlighted [32].

Central Italy

Site	*Locality, Region*	*Altitude (a.s.l.)*	*Chronology*	*Samples*	*Isotopic study*	*Essential references*
Grotta dello Scoglietto	Grosseto, Toscany	20 m	CA-EBA	Humans, 11<br>Animals, 11	33	34–38
Grotta Misa	Viterbo, Lazio	138 m	MBA	Humans, 4<br>Animals, 4	33	35,39–41
Felcetone	Viterbo, Lazio	200 m	MBA	Humans, 12<br>Animals, 0	33	35,40,41
**Grotta Vittorio Vecchi**	**Latina, Lazio**	**505 m**	**MBA**	**Humans, 6<br>Animals, 12**	**this study**	42,43
Grotta Regina Margherita	Frosinone, Lazio	440 m	MBA* (1650-1450 cal. BC)	Humans, 10<br>Animals, 13	44	44,45

*refers to ^{14}C dates. For further information see the relevant references.

Grotta Vittorio Vecchi is a natural 160 m long cave in limestone, at the southern foothills of Fulcino mountain, at the north-east of Sezze (Latina). Discovered in 1987, it is close to the Longara plateau where, during 1960s, several ceramic fragments were recovered and dated to the Middle Bronze Age [46]. Following the archaeological excavation of the funerary cave of Vittorio Vecchi, it has been assumed that the settlement was likely located in Longara and that the associated cemetery was in Gr. Vittorio Vecchi [43]. The cave is composed of some natural chambers which were exploited for different purposes, including burial use and to make offerings [47]. The archaeological remains are diverse: abundant ceramic elements (pots, plates and bowls), bronze tools (rings, daggers and pins), arrowheads, glassy *faïence* and engraved bones [42]. In association with the

archaeological remains, scattered charcoal pieces and the remains of a fireplace were also found. The archaeobotanical investigation on charcoals and seeds identified *Triticum monococcum* and *dicoccum*, *Hordeum volgare*, *Vicia faba* (var. *minuta/equina*) and *Cornus mas* [48]. The archaeobotanical study highlights the particularly high importance of legumes in this site because *Vicia faba* seeds represent 57.26% of the total botanical remains (2182/3811). With regards to the animal remains, domestic and wild animals were recovered in association with the human remains: *Bos taurus*, *Sus domesticus*, *Ovis/Capra*, *Canis* sp., and *Cervus elaphus*. The human remains were rarely found articulated due to both natural post-depositional events, such as landslides, and anthropic activities which disturbed the funerary assemblage. An analysis by Rubini et al. [43] estimated the presence of 40 individuals of both sexes and different ages-at-death, but in this study the isotopic analysis was performed only on four individuals.

Southern Italy and Sicily

Site	*Locality, Region*	*Altitude (a.s.l.)*	*Chronology*	*Samples*	*Isotopic study*	*Essential references*
Lavello	Potenza, Basilicata	300 m	MBA	Humans, 4<br>Animals, 0	18	49
Toppo Daguzzo	Potenza, Basilicata	300 m	MBA	Humans, 21<br>Animals, 0	14 humans – Tafuri et al [18]<br>7 humans – Arena et al. [50]	51,52
Murgia Timone	Matera, Basilicata	425 m	MBA	Humans, 0<br>Animals, 5	50	53,54
**Trinitapoli (Ipogeo dei Bronzi)**	**Madonna di Loreto, Barletta-Andria-Trani, Apulia**	**10 m**	**MBA**	**Humans, 28<br>Animals, 5**	**21 humans – this study<br>7 humans –Arena et al. [50]**	55,56
**Castiglione**	**Ragusa, Sicily**	**50 m**	**EBA**	**Humans, 96<br>Animals, 0**	**this study**	57,58
Punta di Zambrone	Vibo Valentia, Calabria	220 m	RBA	Humans, 2<br>Animals, 15	59	60
Grotta della Monaca	Cosenza, Calabria	740 m	MBA	Humans, 6<br>Animals, 0	50	61,62
Grotta dell’Antenato	Cosenza, Calabria	380 m	MBA	Humans, 1<br>Animals, 0	50	63

Trinitapoli is a site less than 50 kms south-east from Foggia in the region of Daunia, in the north of Apulia. From the 1970s, several artificial hypogea have been found in the area suggesting this region was highly occupied during prehistory. This study included the Ipogeo dei Bronzi, initially used for ritual purposes due to the presence of votive deposits (1850-1700 BC) and later for funerary practices (1700-1300 BC) [56]. This hypogeum is composed of three long corridors, which cover in total almost 50 m, and two areas have been identified (AB and C). More than 200 individuals were inhumed in a collective burial along these corridors, in association with various rich grave goods, mainly composed of bronze objects. Different ornaments depending on the sex and social status of the individuals have been recovered [56]. Females were often buried with rings, earrings, *torques*, bronze pins and also bone *parures*, ivory, precious stones, amber and *faïence* in some particular burials such as that of the “Amber lady”, whose grave goods suggest a prestigious social position for this woman [55,64]. Males were divided into “warriors”, due to their association with different kinds of weapons, such as swords, daggers and arrowheads, and “not-warriors”, with soberer grave goods, mainly represented by objects like buttons [55,65]. Nevertheless, due to the characteristics of the cemetery and some natural post-depositional events such as flooding which disturbed the deposits, individuals generally cannot be directly associated with grave goods, except in some cases [55,56]. The animal remains considered in this study are *Ovis/Capra*, *Canis* sp., and a lagomorph, recovered in association with the human remains. For the interpretation we considered as well two additional individuals (one Cervus elaphus and one caprine) which come from a nearby hypogeum and that have been previously analysed [18]. Several researchers conducted the osteological analysis on the human remains. Here, the assemblage analyzed by Cenni et al. [66] is considered. The individuals come from the two areas of the cemetery (AB and C) and represent both sexes and all ages-at-death, but infants

are rare, probably because they were not usually inhumed with the adults or because the high frailty of their remains led to their bad preservation [67]. Cenni et al. [67] identified 98 individuals, 47 in AB area and 51 in the C area. In the main text the human isotopic data analysed in this research are integrated to seven human data from the same Ipogeum analysed by Arena et al.[50].

Castiglione is a cemetery 7 kms north-west of Ragusa, in the south of Sicily. Since its discovery in 1948, several excavations have revealed a settlement and a cemetery [57,58,68,69]. There are three phases of occupation: the first one in late prehistory and dated to the Early Bronze Age [57,70], the second one in the Archaic period, from VII to V century BC, and the last one during the Classic-Hellenistic period, from IV to III century. Only the settlement was occupied in this last phase [71]. The Early Bronze Age cemetery is composed of seven artificial chamber tombs where archaeological and human remains were closely associated. The grave goods are represented by lithic and bone tools but they mainly consist of pottery as *pithoi* and other different kind of smaller pots like plates and bowls [57,72]. No animal remains were available for analysis. The human remains were organized in collective burials and they are rarely in articulation, probably due to the fact that the chamber tombs were re-opened for subsequent deposits [73]. In total, 175 humans of all ages and both sexes were identified in the seven tombs [74].

Text A.2
State of preservation

Among the human and animal samples, some individuals had a collagen content and C and N elemental compositions outside the accepted ranges and were therefore excluded (Table A.1).
When considering the fauna, four out of 51 samples were excluded due to a low collagen yield (GVV f3, GVV f5, GVV f7 and GVV f12). The yield of extracted collagen for the other samples (n = 47) ranged from 12.9 mg/g to 493.4 mg/g (77.3 mg/g ± 75.6). Elemental composition ranged from 30.7 to 43.7% for carbon (39.1% ± 2.7) and from 10.8 to 15.8 % for nitrogen (14.1% ± 1.0). The carbon to nitrogen (C/N) ratios were between 3.0 and 3.3 (3.2 ± 0.1), falling within the recommended values (DeNiro, 1985).
As for the humans, two individuals from Gr. Vittorio Vecchi, seven from Trinitapoli, 14 from Ostiglia La Vallona and 27 from Castiglione were excluded because of a low collagen yield. One individual, tb 95-10 was excluded by the IRMS analysis because the collagen was visibly very low even though it apparently respected the collagen yield. Individual OLV 100 had a yield very close to the recommended values (9.4 mg/g) and has therefore been considered with caution. One sample from Pertuso and seven samples from Castiglione were excluded because they fell outside the recommended %C, %N or C/N ranges (Table A.1). A Spearman Rho test was applied to detect correlations between the stable isotope values and the preservation criteria. Significant correlations occurred in some cases and particular attention has therefore been devoted to those samples with isotopic results possibly influenced by taphonomic processes (Table A.4).

Text A.3
Comparative analysis: the geographical approach

Northern Italy

The north of Italy includes 13 sites: Pertuso, Buco del Diavolo, Sedegliano, Ostiglia la Vallona, Bovolone, Fondo Paviani, Mereto, Ballabio, Dossetto di Nogara, Olmo di Nogara, Arano di Cellore and Gradisca di Codroipo. In total, 195 humans of different ages-at-death and both sexes and 87 terrestrial and aquatic animals were considered (Table 1).

There is large variation among both human and animal samples (Fig. A.1). Even though the diversity of environments cannot be ignored, these data highlight that northern Italian communities were particularly dynamic in exploiting a variety of resources throughout the entire Bronze Age. The picture is complex but some trends emerge.

Comparing the domestic and wild herbivores and omnivores of each site significant differences occur among these species (Table A.2). These differences can be due to the geography and the landscape of the area where the sites are situated, considering that some sites are in the plain (Arano di Cellore, Olmo di Nogara, Dossetto di Nogara) and others in a mountainous area (Pertuso, Buco del Diavolo, Ballabio). To minimize the environmental differences, the humans have been compared considering the offset between the median of the humans and that of the associated domestic animals for each site to evaluate differences in the diets of human communities (Table A.8).

For the nitrogen values, the enrichment between humans and animals ranges from 2.0‰ at Olmo di Nogara to 4.4‰ at Ostiglia. Given that the typical enrichment between two consecutive trophic levels is between 3 to 5‰ [75,76], it is likely that each site had a different animal protein intake. At Olmo di Nogara, Bovolone and Mereto, the enrichment below 3‰ would suggest a rather limited contribution of animal proteins to the human diet. For all the other sites, it seems animal proteins were more widely consumed. In particular, results support an increase in the consumption of animal protein at the end of the Bronze Age, because Ostiglia (RBA) and Buco del Diavolo (FBA-IA), with values of 4.4‰ and 4.0‰ respectively, show the highest enrichments. In Buco del Diavolo, Liguria, the increased animal product consumption through the Bronze Age becomes more evident when compared to the nearby site of Pertuso (EBA/MBA), which has a lower human-animal offset (3.5‰).

Individuals BA20, OLV371, BV35 and BV43 stand out due to their elevated $\delta^{15}N$ values compared to the rest of their group and to other communities as well. For the juveniles, their ^{15}N enrichment is most likely related to their age (infants) as they were probably still breastfed (OLV371, BV35 and BV43). For the others, the hypotheses explaining their high $\delta^{15}N$ values are mostly linked to pathological and/or social conditions [7,19].

The carbon enrichment between the human groups and the associated fauna is between 0.0‰ and 8‰. This value usually falls between 0‰ and 1‰ [77]. Such high offsets suggest that resources enriched in ^{13}C were regularly consumed by some individuals. Arano (EBA) and Mereto (EBA) both show 0.0‰. Both animals and humans reveal a diet based on C_3 plants, indicating no consumption of C_4 plants, or at least none that can be detected through isotopic analysis. Conversely, the coeval site of Dossetto di Nogara, where the $\Delta^{13}C_{h\text{-}f}$ is 6.9‰, highlights a high enrichment in ^{13}C, supporting the ingestion of C_4 plants by humans. As the animals do not show any C_4 signal, it is likely the humans directly consumed millet [19]. Even if Dossetto is represented by only one individual, this result is exceptional because it provides the first evidence for the regular consumption of C_4 plants in Italy and is dated to the EBA. From this moment on, millets seem to be consumed heterogeneously in the north of the peninsula. In fact, the EBA/MBA sites of Ballabio and Pertuso do not show such enriched values in ^{13}C, implying a diet mainly based on cereals like wheat and barley ($\Delta^{13}C_{h\text{-}f}$ = 0.3‰ and 0.2‰ respectively). Conversely, the coeval humans of Sedegliano show evidence for millet intake, even though the associated animals present a typical C_3 diet ($\Delta^{13}C_{h\text{-}f}$ = 2.5‰). Later MBA/RBA groups, like Bovolone and Olmo di Nogara, show a regular consumption of C_4 plants, with $\Delta^{13}C_{h\text{-}f}$ = 3.5‰ and 5.5‰ respectively. In this case, the high $\Delta^{13}C_{h\text{-}f}$ is once again an indication that millet was probably directly consumed by humans. At the end of the Bronze Age, the consumption of millet seems to be broadly widespread in the north of Italy, as supported by Ostiglia and Buco del Diavolo, where the $\delta^{13}C$ human values are elevated and the $\Delta^{13}C_{h\text{-}f}$ offsets are greater than 1‰ (8.0‰ and 2.1‰ respectively). Thus, this new research confirms that humans directly ate millets because most animals yielded low $\delta^{13}C$, suggesting that the cultivation of this crop was mainly for human food. Only the swine from Fondo Paviani show elevated carbon values; these animals were likely fed food waste [19].

Ostiglia, as widely described in the main text, merits special notice, as it shows both high $\delta^{15}N$ and $\delta^{13}C$ values. As well as terrestrial animal protein and millets, additional aquatic resources may have been consumed.

This site was in a humid environment and the consumption of freshwater animals and some non-nitrogen-fixing plants, all enriched in ^{15}N, cannot be discarded, as attested by the recovery of aquatic remains in the pile-dwelling and Terramare settlements [78]. However, Ostiglia humans do not fall within the ranges of the communities that had a significant consumption of freshwater species, because $\delta^{15}N$ and $\delta^{13}C$ values should be lower [79–81]. Marine species do not seem to have been regularly consumed in this area given the lack of archaeological evidence, as opposed to southern Italy, where these species have been occasionally found [82–84].

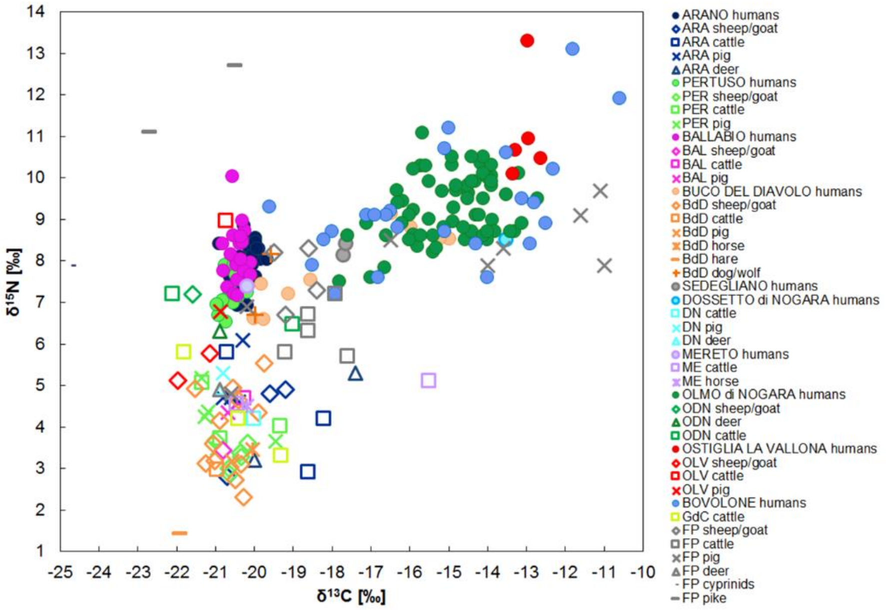

Fig. A.1. $\delta^{15}N$ and $\delta^{13}C$ human and animal values of the sites in northern Italy.

Central Italy

Stable isotope studies in central Italy include five sites for a total of 41 humans and 36 terrestrial animals: Gr. Scoglietto, Gr. Misa, Felcetone, Gr. Regina Margherita and Gr. Vittorio Vecchi.
Kruskal Wallis tests on the animal data show no statistical differences for the $\delta^{13}C$ ratios of the animal groups, contrary to the $\delta^{15}N$ ratios (Table A.5). Nevertheless, these results mostly overlap for both carbon and nitrogen values, indicating that most of the resources originated from a C_3 terrestrial temperate environment and that the sites were situated in areas with similar ecological characteristics.

Since the values for the animals are consistent, the human groups can be directly compared. Statistically significant differences for both $\delta^{15}N$ and $\delta^{13}C$ values exist and Wilcoxon-Mann-Whitney tests for each pair of sites show that differences in nitrogen values occur between all pairs, except Gr. Vittorio Vecchi and Felcetone and Gr. Regina Margherita and Felcetone (Table A.6), this because some individuals of the three communities overlap, likely suggesting a similar animal protein intake. For carbon results, Gr. Regina Margherita show the lowest $\delta^{13}C$ values, in fact it statistically differs from all the other sites, as well as Gr. Scoglietto and Gr. Misa have significant differences. Consequently, the dietary choices of the humans were diverse and different patterns can be identified. Gr. Scoglietto has higher $\delta^{15}N$ values compared with its fauna ($\Delta^{15}N_{h\text{-}f}$ = 4.7‰) and the other human groups and it is likely that individuals consumed terrestrial animal protein as well as aquatic resources [33]. Gr. Scoglietto is placed on the Tyrrenian coast and at that time it was in lagoon-like area: this environment may have led the population to exploit local aquatic resource at the expense of agricultural products. Even though dental calculus analysis corroborates the consumption of cereals like millets [85], the isotopic data do not indicate that these plants were commonly eaten. Furthermore, the dental calculus results support the absence of any

leguminous species [85]. The apparent absence of these plants in the diet associated to an important ingestion of animal foodstuffs can reasonably explain the enrichment in ^{15}N.

Individuals from Gr. Misa had a mixed terrestrial diet of both animal and vegetal resources. In addition, δ^{13}C ratios denote an important consumption of plant protein at Gr. Misa [33] and δ^{13}C values higher than -18‰ suggest that some individuals ate C_4 plants. Furthermore, remains of broomcorn millet have been found in the cave [86]. The humans from Felcetone, a few kilometers from Gr. Misa, also had a mixed terrestrial diet, but, in this case, δ^{13}C and δ^{15}N data indicate a more important plant consumption, with a significant contribution of C_4 plants in some individuals. As for the δ^{15}N values, these are relatively low when compared with the local fauna (cf. Gr. Misa samples), implying a low animal protein intake alongside legume consumption. Gr. Regina Margherita show a low animal protein intake too, but the lowest δ^{13}C ratios support a diet uniquely based on C_3 plants. Only one individual shows a particular high δ^{15}N value outside the local range likely because still breastfed [44]. Finally, the individuals from Gr. Vittorio Vecchi show a different pattern compared with the previous communities. The animal protein intake was a minor component of the diet and, on the contrary, C_3 plants and legumes seem to have been significantly consumed (Fig. A.2).

Therefore, the five human groups of the central Italy, even if some of them present a small sample size, show different food habits. According to the local animal results that are homogenous, this variability can be attributed to different choices and not to different environments. The assortment of resources is evident at an intra- and inter-population level, except for Gr. Regina Margherita and Gr. Vittorio Vecchi, where the results reveal an intra-group homogeneous diet.

Chronologically, these data indicate greater legume (Felcetone, Gr. Vittorio Vecchi) and likely millet (Gr. Misa, Felcetone) consumption and a lower animal protein intake (Felcetone, Gr. Misa, Gr. Vittorio Vecchi, Gr. Regina Margherita) in the MBA when compared with the EBA site (Gr. Scoglietto).

The different food habits recorded in this area may have resulted from the interaction of multiple causes. It is likely that the varied geomorphology of the region favored some crops, like pulses or millets, orienting the agricultural practices towards them. Moreover, the heterogeneity of the dietary trends reflects the capacity of each community to select the most suitable form of subsistence practices and crops according to its needs. Despite the more frequent contacts between local communities, this phenomenon shows how each group tended to preserve its food traditions. Further analyses of coeval local sites are needed to better understand the specificities of the area.

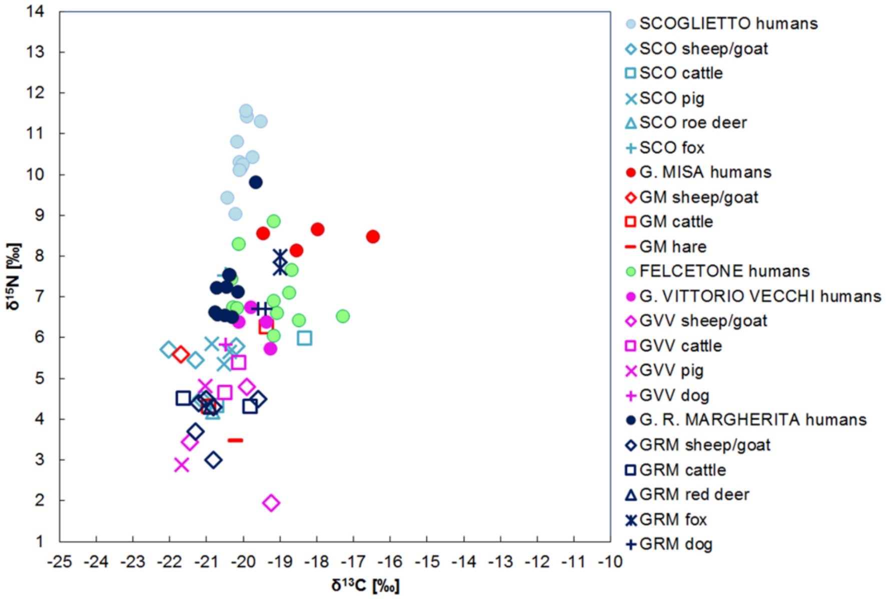

Fig. A.2. The δ^{15}N and δ^{13}C human and animal values of the sites in central Italy.

Southern Italy

The study includes eight sites from Apulia, Basilicata, Calabria and Sicily: Trinitapoli, Lavello, Toppo Daguzzo, Punta di Zambrone, Murgia Timone, Gr. della Monaca, Gr. dell'Antenato and Castiglione, for a total of 116 humans and 25 terrestrial and aquatic animals.

The fauna comes from Trinitapoli, Murgia Timone and Punta di Zambrone. Significant statistical differences occur for both carbon and nitrogen terrestrial animal values (Table A.5). These are attributed to the fact that Trinitapoli and Murgia Timone specimens indicate a C_3 diet. On the contrary, Punta di Zambrone animals present a heterogeneous diet, composed of C_3 and C_4 plants. Indeed, animals from the latter site show a wide $\delta^{13}C$ variability, spreading from -20.6‰ to -14.9‰.

Given the differences between the animal assemblages and the little number of humans at Punta di Zambrone, this site is considered separately. First only humans from Toppo Daguzzo, Lavello, Trinitapoli and Gr. della Monaca have been considered. Kruskal-Wallis tests show no significant differences among these sites for nitrogen ($p = 0.629$), contrary to $\delta^{13}C$ ($p = 0.035$) (Table A.6). The latter is likely due to slight regional differences. Nevertheless, the median of the four groups is similar and the $\delta^{15}N$ and $\delta^{13}C$ ranges are narrow and, for the most part, overlap, suggesting a homogeneous diet at an intra- and interpopulation level (Fig. A.3). The individual of Gr. dell'Antenato, not included in the statistical analysis, is consistent with these data, fitting in the N and C ranges. This result suggests a consistent dietary pattern, mainly based on C_3 terrestrial foodstuffs. The $\Delta^{15}N_{h-f}$ between the humans and the local animals are lower than those normally found between two consecutive trophic levels (Table A.8). This indicate a minor animal protein intake and a likely important contribution of legumes in the diet, as also supported by the gradual increase in legume recovery rate recorded from the Bronze Age to the Iron Age in local archeobotanical studies [87,88]. Species such as beans, lentils and probably vetches were commonly cultivated, thus the consumption of these plants may have contributed to the low nitrogen values. Research on Coppa Nevigata humans seems also to confirm this trend [89].
All the funerary sites are coeval and they are located just a few kilometers away from each other. Given the similar isotopic values, it is likely that they all had a similar environment. Palynological and carpological studies conducted on nearby sites suggest a predominantly open environment, with widespread grasslands from the Bronze Age, in response to a climate shift towards aridity and seasonal variability in temperature and rainfall patterns, which affected the vegetation cover [88,90,91]. The results can therefore be the consequence of identical agricultural practices in a similar environment as well as that of frequent exchanges between the local human groups, resulting in the analogous diet of local communities.

When comparing Castiglione humans with the other groups in southern Italy, differences emerge. Kruskal-Wallis tests for Trinitapoli, Toppo Daguzzo, Lavello, Gr. della Monaca and Castiglione show significant differences for both nitrogen and carbon values (Table A.7). For the nitrogen, this is due to different $\delta^{15}N$ ranges. Even if they overlap, the lower median of Castiglione (7.5‰) compared with the one of Trinitapoli, Toppo Daguzzo, Lavello and Gr. della Monaca (8.3‰) clearly underscores this variability. It is likely that the Castiglione humans had a more varied diet, mainly linked to a diverse, direct or indirect animal protein intake. Concerning carbon results, Castiglione human $\delta^{13}C$ values are more enriched in ^{13}C than those from the other groups (Table A.3), suggesting the consumption of plants with higher $\delta^{13}C$. Such an enrichment could be the result of different environmental conditions influencing carbon values from the lowest level in the trophic chain. This is supported by a number of isotopic studies which show how altitude, salinity, temperature and dryness of the soil, mainly related to the frequency and amount of rainfall, affect plant $\delta^{13}C$ values [92–95]. In addition, the lower latitude can also positively influence $\delta^{13}C$ values [96]. Despite this, food patterns support a diet mainly based on C_3 resources for the entire south of the peninsula, including Sicily, with no substantial discrepancies until the end of the MBA. The only evidence for C_4 plant consumption in the south of Italy is suggested by Rumolo et al.

59 for the site of Punta di Zambrone (RBA). This seems to be the earliest signal for millet intake in the south of Italy, later than in the north and in central Italy.

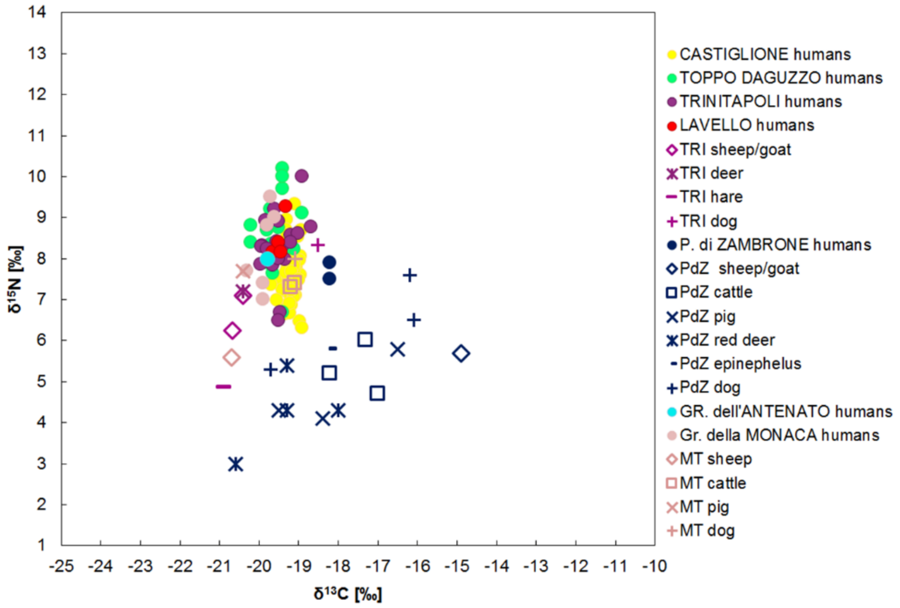

Fig. A.3. The $\delta^{15}N$ and $\delta^{13}C$ human and animal values of the sites in southern Italy.

Table A.1
The sample dataset

The samples were taken from sites with single and multiple burials. For individual burials the selection was preferentially carried out on fragmented bones. In the collective burials and in the absence of articulated remains, only elements used to estimate the MNI (Minimum Number of Individuals) were sampled.
Sex and age-at-death, when evaluated through an osteological study, like in the case of Ostiglia La Vallona [97], were reported as such. For the sites where no information was available, for consistency, the same methods as in the other sites were applied.
Sex diagnosis was carried out on the cranium, the mandible, pelvic morphology and metric assessments [98–103]. The humerus and femur were used in a few cases [104,105].
Age-at-death was estimated through tooth wear and dental development [106–110] and from the postcranial bones [111]. When an age assessment was not possible, the known age-at-death skeletal infant collection preserved at the Laboratory of Anthropology of the University of Florence was used for comparison. Due to the differential accuracy of the methods, the individuals were divided into the following age categories: infant = 0-4 yrs; child = 5-15 yrs; adolescent = 16-19 yrs; young adult = 20-29 yrs; adult > 30 yrs.

Results of stable carbon and nitrogen isotopes for the selected sites (infant = 0-4 yrs; child = 5-15 yrs; adolescent = 16-19 yrs; young adult = 20-29 yrs; adult > 30 yrs; N.D. = not defined, M = male, F = female. In grey the samples excluded from the study).

ID	*Site*	Species	*Age*	*Sex*	*Burial*	*Rdt (mg/g)*	*% N*	*$\delta^{15}N_{AIR}$ (‰)*	*% C*	*$\delta^{13}C_{V-PDB}$ (‰)*	*C/N*
Per A	Pertuso	human	adult	N.D.	-	97.8	14.6	7.1	40.7	-20.4	3.2
Per B	Pertuso	human	adult	N.D.	-	114.9	14.3	7.2	39.9	-20.2	3.3
Per C	Pertuso	human	adult	N.D.	-	76.1	13.8	7.9	38.7	-20.7	3.3
Per E	Pertuso	human	adult	N.D.	-	38.8	14.5	7.7	40.1	-20.5	3.2
Per F	Pertuso	human	adult	N.D.	-	123.5	14.1	7.2	39.3	-20.6	3.3
Per G	Pertuso	human	adult	N.D.	-	65.8	14.3	7.9	39.5	-20.5	3.2
Per H	Pertuso	human	adult	N.D.	-	47.2	14.9	7.7	41.6	-20.5	3.3
Per I	Pertuso	human	adult	N.D.	-	42.1	12.5	7.0	35.0	-20.5	3.3
Per L	Pertuso	human	child-adolescent (14-15 yrs)	N.D.	-	50.9	14.1	6.5	38.5	-20.7	3.2
Per Ab	Pertuso	human	child (6-7 yrs)	N.D.	-	80.2	13.7	6.7	38.4	-20.9	3.3
Per Bb	Pertuso	human	child (6 yrs)	N.D.	-	67.0	11.7	7.0	33.4	-20.8	3.3
Per Cb	Pertuso	human	child (5-6 yrs)	N.D.	-	52.7	14.2	7.0	39.1	-21.0	3.2
Per Db	Pertuso	human	child (5-6 yrs)	N.D.	-	77.5	14.1	7.2	38.8	-20.6	3.2
Per D	Pertuso	human	adult	N.D.	-	38.6	10.8	6.8	57.1	-26.5	6.1
Per A o/c	Pertuso	sheep/goat	-	-	-	35.3	14.8	3.7	41.1	-21.0	3.2
Per B o/c	Pertuso	sheep/goat	-	-	-	69.9	15.2	3.3	41.6	-20.3	3.2
Per C o/c	Pertuso	sheep/goat	-	-	-	87.8	14.7	3.4	40.5	-20.4	3.2
Per D o/c	Pertuso	sheep/goat	-	-	-	39.9	14.8	2.9	40.9	-20.6	3.2
Per E o/c	Pertuso	sheep/goat	-	-	-	19.8	14.5	3.6	40.5	-20.2	3.2
Per F o/c	Pertuso	sheep/goat	-	-	-	22.6	14.6	3.0	40.4	-20.7	3.2
Per G sus	Pertuso	pig	-	-	-	19.2	14.0	3.7	39.0	-19.5	3.2
Per H sus	Pertuso	pig	-	-	-	61.1	14.4	4.2	39.8	-21.3	3.2
Per I sus	Pertuso	pig	-	-	-	36.5	14.9	5.2	41.4	-21.4	3.2
Per L sus	Pertuso	pig	-	-	-	12.9	14.0	3.5	38.3	-21.0	3.2
Per M sus	Pertuso	pig	-	-	-	26.2	14.4	4.4	40.0	-21.2	3.3
Per N bos	Pertuso	cattle	-	-	-	43.7	13.3	4.0	36.2	-19.3	3.2

ID	*Site*	**Species**	*Age*	*Sex*	*Burial*	*Rdt (mg/g)*	*% N*	$\delta^{15}N_{AIR}$ (‰)	*% C*	$\delta^{13}C_{V-PDB}$ (‰)	*C/N*
Per O bos	Pertuso	cattle	-	-	-	44.3	14.9	5.1	41.1	-21.3	3.2
Per P bos	Pertuso	cattle	-	-	-	22.0	14.5	3.7	39.9	-20.9	3.2
BD A	Buco del Diavolo	human	adult	N.D.	-	90.7	14.8	6.6	41.2	-20.0	3.3
BD 716	Buco del Diavolo	human	adult	M	-	69.8	14.7	7.4	40.3	-19.8	3.2
BD 1320	Buco del Diavolo	human	adolescent (16-19 yrs)	N.D.	-	89.9	14.9	7.2	41.1	-19.1	3.2
BD 876	Buco del Diavolo	human	adult	M	-	21.1	14.5	8.8	40.4	-16.0	3.3
BD 1092	Buco del Diavolo	human	young adult	N.D.	-	158.6	15.2	7.5	41.5	-18.5	3.2
BD 1132	Buco del Diavolo	human	young adult	F	-	51.1	14.3	9.1	39.6	-16.5	3.2
BD 882	Buco del Diavolo	human	adolescent (16-19 yrs)	M	-	90.9	15.1	8.5	41.8	-15.0	3.2
BD 794	Buco del Diavolo	human	adult	M	-	56.4	15.3	8.5	42.1	-15.2	3.2
BD 2453	Buco del Diavolo	human	child (10 yrs)	N.D.	-	67.6	14.6	6.6	40.0	-19.8	3.2
BD f 19857 o/c	Buco del Diavolo	sheep/goat	-	-	-	153.3	14.8	2.3	40.5	-20.3	3.2
BD f 20147 (b) o/c	Buco del Diavolo	sheep/goat	-	-	-	163.8	14.6	3.1	40.3	-20.3	3.2
BD f 20151 o/c	Buco del Diavolo	sheep/goat	-	-	-	92.3	14.6	3.1	40.7	-21.3	3.3
BD f 349 o/c	Buco del Diavolo	sheep/goat	-	-	-	156.9	14.5	5.5	40.3	-19.8	3.2
BD f 20197 o/c	Buco del Diavolo	sheep/goat	-	-	-	60.5	13.8	5.0	39.1	-20.6	3.3
BD f 19846 o/c	Buco del Diavolo	sheep/goat	-	-	-	103.1	13.5	2.7	37.9	-20.5	3.3
BD f 19878 o/c	Buco del Diavolo	sheep/goat	-	-	-	493.4	14.4	3.6	40.4	-21.1	3.3
BD f 764 o/c	Buco del Diavolo	sheep/goat	-	-	-	64.6	13.4	3.2	36.9	-21.0	3.2
BD f 362 o/c	Buco del Diavolo	sheep/goat	-	-	-	43.9	10.8	4.9	30.7	-21.5	3.3
BD f 20147 (a) o/c	Buco del Diavolo	sheep/goat	-	-	-	133.6	14.6	4.1	40.1	-20.9	3.2
BD f 311 o/c	Buco del Diavolo	sheep/goat	-	-	-	46.5	14.6	4.6	40.5	-20.4	3.2
BD f 20153 o/c	Buco del Diavolo	sheep/goat	-	-	-	147.6	14.8	4.4	41.2	-19.9	3.3
BD f 20350 canis	Buco del Diavolo	dog	-	-	-	94.1	14.5	8.2	40.4	-19.6	3.2
BD f 20353 canis	Buco del Diavolo	dog	-	-	-	65.9	14.4	6.7	39.7	-20.0	3.2
BD f 20209 bos	Buco del Diavolo	cattle	-	-	-	100.0	15.3	3.0	41.2	-20.9	3.1
BD f 20267 equus	Buco del Diavolo	horse	-	-	-	90.7	12.9	3.5	36.2	-20.0	3.3
BD f 712 lepus	Buco del Diavolo	hare	-	-	-	42.7	14.0	1.4	38.9	-21.9	3.3
BD f 20198 sus	Buco del Diavolo	pig	-	-	-	78.4	13.9	3.2	38.0	-20.6	3.2
OLV 183	Ostiglia La Vallona	human	adolescent (18 yrs)	M	Tb. 183	15.5	11.1	10.1	31.5	-13.3	3.3
OLV 371 sub	Ostiglia La Vallona	human	infant (1-1.5 yrs)	N.D.	Tb. 371	9.8	11.3	13.3	32.3	-13.0	3.3
OLV 189	Ostiglia La Vallona	human	child (11-12 yrs)	N.D.	Tb. 189	11.9	13.1	10.5	35.9	-12.6	3.2
OLV 359	Ostiglia La Vallona	human	Infant/child (4 yrs ± 12 mth)	N.D.	Tb. 359	10.8	12.9	10.9	36.4	-12.9	3.3
OLV 100	Ostiglia La Vallona	human	adult	N.D.	Tb. 100	9.4	12.7	10.7	35.7	-13.3	3.3
OLV F bos	Ostiglia La Vallona	cattle	-	-	-	130.0	15.8	9.0	43.7	-20.7	3.2
OLV F c1	Ostiglia La Vallona	sheep/goat	-	-	-	127.7	14.5	5.1	40.4	-22.0	3.2
OLV F c2	Ostiglia La Vallona	sheep/goat	-	-	-	126.1	14.1	5.8	39.4	-21.2	3.3
OLV F sus	Ostiglia La Vallona	pig	-	-	-	115.7	14.6	6.8	40.8	-20.9	3.3
OLV 361	Ostiglia La Vallona	human	child (12-13 yrs)	N.D.	Tb. 361	1.0	-	-	-	-	-
OLV 181	Ostiglia La Vallona	human	child (5-6 yrs)	N.D.	Tb. 181	2.3	-	-	-	-	-
OLV 111	Ostiglia La Vallona	human	adult	F	Tb. 111	0.0	-	-	-	-	-
OLV 188	Ostiglia La Vallona	human	young adult (25-30 yrs)	F	Tb. 188	0.3	-	-	-	-	-
OLV 101	Ostiglia La Vallona	human	adult (35-40 yrs)	F	Tb. 101	8.4	-	-	-	-	-
OLV 379	Ostiglia La Vallona	human	child (10-11 yrs)	N.D.	Tb. 379	3.3	-	-	-	-	-
OLV 371 ad.	Ostiglia La Vallona	human	young adult (20-23 yrs)	F	Tb. 371	5.2	-	-	-	-	-
OLV 17	Ostiglia La Vallona	human	adult (40-45 yrs)	M	Tb. 17	24.6	9.3	9.7	28.0	-16.4	3.5
OLV 95	Ostiglia La Vallona	human	young adult (25-30 yrs)	M	Tb. 95	2.0	-	-	-	-	-
OLV 176	Ostiglia La Vallona	human	young adult	F	Tb. 176	0.3	-	-	-	-	-

ID	***Site***	**Species**	***Age***	***Sex***	***Burial***	***Rdt (mg/g)***	***% N***	***$\delta^{15}N_{AIR}$ (‰)***	***% C***	***$\delta^{13}C_{V\text{-}PDB}$ (‰)***	***C/N***
			(20-25 yrs)								
OLV 378	Ostiglia La Vallona	human	adult (35-40 yrs)	M	Tb. 378	4.9	-	-	-	-	-
OLV 183 sub	Ostiglia La Vallona	human	child (10-11 yrs)	N.D.	Tb. 183	0.3	-	-	-	-	-
OLV 99	Ostiglia La Vallona	human	adult (40-45 yrs)	M	Tb. 99	4.0	-	-	-	-	-
OLV 96	Ostiglia La Vallona	human	child (9-10 yrs)	N.D.	Tb. 96	1.6	-	-	-	-	-
S 7°	Grotta Vittorio Vecchi	human	child (13-15 yrs)	N.D.		79.7	14.8	5.7	41.5	-19.2	3.3
S 7	Grotta Vittorio Vecchi	human	adult	N.D.		38.3	13.6	6.7	38.7	-19.8	3.3
S 16	Grotta Vittorio Vecchi	human	adult	N.D.		10.9	12.7	6.4	36.6	-19.4	3.4
S 12	Grotta Vittorio Vecchi	human	adult	N.D.		28.5	14.0	6.4	39.6	-20.1	3.3
S 27	Grotta Vittorio Vecchi	human	adult	N.D.		8.8	-	-	-	-	-
S 11b	Grotta Vittorio Vecchi	human	adult	N.D.		2.7	-	-	-	-	-
GVV f 1	Grotta Vittorio Vecchi	dog	-	-		27.7	11.1	5.8	30.7	-20.5	3.2
GVV f 2	Grotta Vittorio Vecchi	sheep/goat	-	-		17.9	13.5	1.9	34.2	-21.5	3.0
GVV f 4	Grotta Vittorio Vecchi	cattle	-	-		39.8	14.4	4.6	40.0	-20.5	3.2
GVV f 6	Grotta Vittorio Vecchi	sheep/goat	-	-		22.7	13.6	3.4	38.1	-19.9	3.3
GVV f 8	Grotta Vittorio Vecchi	pig	-	-		27.6	11.6	4.8	33.1	-21.0	3.3
GVV f 9	Grotta Vittorio Vecchi	pig	-	-		52.37	13.8	2.9	38.1	-21.7	3.2
GVV f 10	Grotta Vittorio Vecchi	sheep/goat	-	-		42.1	14.3	4.8	39.2	-19.2	3.2
GVV f 11	Grotta Vittorio Vecchi	cattle	-	-		39.7	12.7	5.4	35.8	-20.1	3.3
GVV f 12	Grotta Vittorio Vecchi	dog	-			0.3	-	-	-	-	-
GVV f 3	Grotta Vittorio Vecchi	pig	-			5.9	-	-	-	-	-
GVV f 5	Grotta Vittorio Vecchi	red deer	-			2.7	-	-	-	-	-
GVV f 7	Grotta Vittorio Vecchi	pig	-			5.2	-	-	-	-	-
TRI AB 20	Trinitapoli	human	adult	F	Area AB	48.3	14.3	6.7	39.2	-19.5	3.2
TRI AB 22	Trinitapoli	human	adult	F	Area AB	16.1	14.1	6.5	36.6	-19.5	3.0
TRI AB 4	Trinitapoli	human	adult	F	Area AB	68.7	13.9	8.3	39.9	-19.4	3.3
TRI AB 16	Trinitapoli	human	adult	M	Area AB	18.7	11.6	8.4	33.1	-19.5	3.3
TRI AB 35	Trinitapoli	human	adult	M	Area AB	83.2	15.4	7.8	42.4	-19.7	3.2
TRI AB 13	Trinitapoli	human	adult	M	Area AB	42.0	14.4	8.3	39.8	-19.9	3.2
TRI AB 6	Trinitapoli	human	adult	M	Area AB	41.0	13.8	8.9	40.2	-19.8	3.4
TRI C 94	Trinitapoli	human	adult	F	Area C	40.1	15.1	7.9	41.6	-20.0	3.2
TRI C 98	Trinitapoli	human	adult	F	Area C	67.1	14.5	8.3	41.5	-19.9	3.3
TRI C 93	Trinitapoli	human	adult	F	Area C	37.0	14.0	8.6	38.5	-19.2	3.2
TRI C 115	Trinitapoli	human	adult	M	Area C	58.2	13.8	8.1	38.4	-19.5	3.2
TRI C 194	Trinitapoli	human	adult	M	Area C	54.4	15.2	8.8	42.0	-18.7	3.2
TRI C 92	Trinitapoli	human	adult	M	Area C	12.0	13.0	8.2	36.5	-19.8	3.3
TRI C 99	Trinitapoli	human	adult	M	Area C	49.9	13.7	8.0	37.4	-19.3	3.2
TRI AB 24	Trinitapoli	human	adult	M	Area AB	0.0	-	-	-	-	-
TRI AB 30	Trinitapoli	human	adult	F	Area AB	0.0	-	-	-	-	-
TRI AB 10	Trinitapoli	human	adult	F	Area AB	1.0	-	-	-	-	-
TRI AB 23	Trinitapoli	human	adult	F	Area AB	1.0	-	-	-	-	-
TRI C 103	Trinitapoli	human	adult	M	Area C	0.0	-	-	-	-	-
TRI C 96	Trinitapoli	human	adult	F	Area C	8.0	-	-	-	-	-
TRI C 100	Trinitapoli	human	adult	F	Area C	2.6	-	-	-	-	-
TRI F o/c	Trinitapoli	sheep/goat	-	-	-	67.3	14.7	6.2	41.1	-20.7	3.3
TRI F log	Trinitapoli	hare	-	-	-	104.3	14.6	4.9	40.7	-20.9	3.2
TRI F can	Trinitapoli	dog	-	-	-	19.6	13.8	8.3	38.7	-18.5	3.3
**Tb. 93 C**	Castiglione	human	adult	N.D.	Tb. 93	22.6	12.4	7.8	35.9	-19.3	3.4
**Tb. 93 F**	Castiglione	human	adult	N.D.	Tb. 93	44.6	12.9	7.8	36.5	-19.0	3.3
**Tb. 93 H**	Castiglione	human	adult	N.D.	Tb. 93	32.9	13.2	8.5	37.5	-19.2	3.3

ID	Site	Species	Age	Sex	Burial	Rdt (mg/g)	% N	$\delta^{15}N_{AIR}$ (‰)	% C	$\delta^{13}C_{V\text{-}PDB}$ (‰)	C/N
**Tb. 93 G**	Castiglione	human	adult	F	Tb. 93	11.4	10.6	8.4	31.0	-19.5	3.4
**Tb. 93-10**	Castiglione	human	adult	N.D.	Tb. 93	44.4	14.1	8.7	40.0	-18.9	3.3
**Tb. 93 B**	Castiglione	human	adult	N.D.	Tb. 93	26.7	13.1	7.4	38.1	-19.4	3.4
**Tb. 93-4**	Castiglione	human	adult	N.D.	Tb. 93	40.8	14.3	7.5	40.4	-19.1	3.3
**Tb. 93-8**	Castiglione	human	adult	N.D.	Tb. 93	20.0	14.6	7.2	41.4	-19.3	3.3
**Tb. 93-7**	Castiglione	human	adult	N.D.	Tb. 93	45.2	14.2	7.6	40.4	-18.9	3.3
**Tb. 93 A**	Castiglione	human	adult	M	Tb. 93	13.9	11.3	8.0	32.4	-19.5	3.3
**Tb. 93 I**	Castiglione	human	child (5,5-6,5 yrs)	N.D.	Tb. 93	20.2	15.8	7.5	44.5	-19.5	3.3
**Tb. 93-50**	Castiglione	human	adult	N.D.	Tb. 93	26.1	14.3	8.0	41.0	-19.3	3.3
**Tb. 93 E**	Castiglione	human	adult	N.D.	Tb. 93	45.2	14.8	7.7	42.0	-19.1	3.3
**Tb. 93-15**	Castiglione	human	adult	N.D.	Tb. 93	67.3	14.6	7.3	41.3	-19.2	3.3
**Tb 93-21**	Castiglione	human	adult	N.D.	Tb. 93	1.3	-	-	-	-	-
**Tb 93 D**	Castiglione	human	adult	N.D.	Tb. 93	5.2	-	-	-	-	-
**Tb 93-62**	Castiglione	human	adult	N.D.	Tb. 93	3.3	-	-	-	-	-
**Tb 93-68**	Castiglione	human	adult	N.D.	Tb. 93	3.6	-	-	-	-	-
**Tb 93-16**	Castiglione	human	adult	N.D.	Tb. 93	5.2	-	-	-	-	-
**Tb 93-13**	Castiglione	human	adult	N.D.	Tb. 93	1.3	-	-	-	-	-
**Tb. 119-48**	Castiglione	human	adult	N.D.	Tb. 119	29.7	14.7	7.9	42.1	-19.1	3.3
**Tb. 119-76**	Castiglione	human	adult	N.D.	Tb. 119	39.4	14.8	7.3	42.0	-19.1	3.3
**Tb. 119-12**	Castiglione	human	adult	N.D.	Tb. 119	77.6	14.0	8.1	39.5	-19.0	3.3
**Tb. 119-7**	Castiglione	human	adult	N.D.	Tb. 119	17.2	12.0	8.3	34.5	-19.3	3.4
**Tb. 119-61**	Castiglione	human	adult	N.D.	Tb. 119	19.3	13.1	8.0	37.4	-19.3	3.3
**Tb. 119-82**	Castiglione	human	adult	N.D.	Tb. 119	10.5	13.4	7.5	38.9	-19.3	3.4
**Tb. 119-78**	Castiglione	human	adult	N.D.	Tb. 119	48.2	13.7	8.2	39.1	-19.4	3.3
**Tb. 119-67**	Castiglione	human	adult	N.D.	Tb. 119	22.3	14.9	7.2	43.2	-19.4	3.4
**Tb. 119-4**	Castiglione	human	adult	N.D.	Tb. 119	14.6	14.7	8.9	43.0	-19.3	3.4
**Tb. 119-2**	Castiglione	human	adult	N.D.	Tb. 119	10.8	9.2	7.4	26.5	-19.3	3.3
**Tb. 119-37**	Castiglione	human	adult	N.D.	Tb. 119	13.7	10.7	8.2	31.5	-19.0	3.4
**Tb. 119-35**	Castiglione	human	adult	N.D.	Tb. 119	25.5	17.2	8.0	48.4	-19.0	3.3
**Tb. 119-26**	Castiglione	human	adult	N.D.	Tb. 119	21.5	14.8	8.7	42.5	-19.3	3.3
**Tb 119-3**	Castiglione	human	adult	N.D.	Tb. 119	2.7	-	-	-	-	-
**Tb 119-13**	Castiglione	human	adult	N.D.	Tb. 119	8.8	-	-	-	-	-
**Tb 119-31**	Castiglione	human	adult	N.D.	Tb. 119	7.9	-	-	-	-	-
**Tb 119-24**	Castiglione	human	adult	N.D.	Tb. 119	0.0	-	-	-	-	-
**Tb 119-46**	Castiglione	human	adult	N.D.	Tb. 119	4.5	-	-	-	-	-
**Tb 119-79**	Castiglione	human	adult	N.D.	Tb. 119	3.3	-	-	-	-	-
**Tb 119-6**	Castiglione	human	adult	N.D.	Tb. 119	0.6	-	-	-	-	-
**Tb 119-21**	Castiglione	human	adult	N.D.	Tb. 119	4.0	-	-	-	-	-
**Tb 119-55**	Castiglione	human	adult	N.D.	Tb. 119	8.8	-	-	-	-	-
**Tb 119-25**	Castiglione	human	adult	N.D.	Tb. 119	3.9	-	-	-	-	-
**Tb 119-29**	Castiglione	human	adult	N.D.	Tb. 119	1.0	-	-	-	-	-
**Tb. 95-21**	Castiglione	human	adult	N.D.	Tb. 95	50.6	14.4	6.9	39.8	-19.2	3.2
**Tb. 95-28**	Castiglione	human	adult	N.D.	Tb. 95	80.3	14.8	7.6	40.8	-19.2	3.2
**Tb. 95-43**	Castiglione	human	adult	N.D.	Tb. 95	34.6	15.0	7.1	41.3	-19.1	3.2
**Tb. 95-33**	Castiglione	human	adult	N.D.	Tb. 95	35.8	14.4	6.8	39.8	-19.3	3.2
**Tb. 95-49**	Castiglione	human	child (6 yrs)	N.D.	Tb. 95	16.7	12.6	6.5	35.1	-19.0	3.3
**Tb. 95-11**	Castiglione	human	adult	M	Tb. 95	27.8	12.9	7.4	35.3	-19.1	3.2
**Tb. 95-24**	Castiglione	human	adult	N.D.	Tb. 95	62.1	14.1	7.9	39.2	-19.1	3.2
**Tb. 95-A**	Castiglione	human	adult	N.D.	Tb. 95	31.3	13.9	7.4	38.8	-19.2	3.3
**Tb. 95 D**	Castiglione	human	adult	N.D.	Tb. 95	92.3	13.9	6.6	38.8	-19.3	3.3
**Tb. 95 F**	Castiglione	human	adult	N.D.	Tb. 95	44.7	13.6	7.3	37.7	-19.2	3.2

ID	Site	Species	Age	Sex	Burial	Rdt (mg/g)	% N	$\delta^{15}N_{AIR}$ (‰)	% C	$\delta^{13}C_{V\text{-}PDB}$ (‰)	C/N
**Tb. 95 H**	Castiglione	human	adult	F	Tb. 95	29.5	13.7	7.4	37.9	-19.1	3.2
**Tb. 95-B**	Castiglione	human	adult	N.D.	Tb. 95	33.7	13.0	7.9	36.5	-19.1	3.3
**Tb. 95-19**	Castiglione	human	adult	N.D.	Tb. 95	12.0	10.5	6.7	33.0	-20.7	3.7
**Tb. 95-18**	Castiglione	human	adult	N.D.	Tb. 95	38.3	15.2	7.4	43.1	-19.2	3.3
**Tb. 95-16**	Castiglione	human	adult	F	Tb. 95	31.3	12.5	7.0	35.6	-19.6	3.3
**Tb. 95-10**	Castiglione	human	adult	N.D.	Tb. 95	28.1	-	-	-	-	-
**Tb 95-41**	Castiglione	human	adult	N.D.	Tb. 95	0.3	-	-	-	-	-
**Tb 95 26**	Castiglione	human	adult	N.D.	Tb. 95	0.0	-	-	-	-	-
**Tb 95 C**	Castiglione	human	adult	N.D.	Tb. 95	4.9	-	-	-	-	-
**Tb 95 E**	Castiglione	human	adult	N.D.	Tb. 95	2.3	-	-	-	-	-
**Tb 95 G**	Castiglione	human	adult	N.D.	Tb. 95	0.0	-	-	-	-	-
**Tb. 114 B**	Castiglione	human	child (5,5-6,5 yrs)	N.D.	Tb. 114	11.3	14.6	3.4	35.8	-19.1	2.9
**Tb. 114 -1**	Castiglione	human	child (7 yrs)	N.D.	Tb. 114	76.6	14.1	7.1	39.2	-19.2	3.2
**Tb. 114-A4**	Castiglione	human	adult	N.D.	Tb. 114	16.8	10.7	3.7	26.7	-19.9	2.9
**Tb. 114-4**	Castiglione	human	adult	N.D.	Tb. 114	23.8	13.8	7.1	38.9	-19.1	3.3
**Tb. 114-6**	Castiglione	human	adult	N.D.	Tb. 114	31.7	14.1	6.9	39.4	-19.2	3.3
**Tb. 114-8**	Castiglione	human	adult	N.D.	Tb. 114	14.7	12.9	8.6	36.8	-19.3	3.3
**Tb. 114-9**	Castiglione	human	adult	N.D.	Tb. 114	19.2	14.0	7.7	39.0	-19.2	3.3
**Tb. 114-11**	Castiglione	human	adult	N.D.	Tb. 114	23.0	13.9	7.8	39.5	-19.1	3.3
**Tb. 94-2**	Castiglione	human	adult	N.D.	Tb. 94	27.0	13.7	8.5	38.5	-19.0	3.3
**Tb. 94-5**	Castiglione	human	adult	N.D.	Tb. 94	21.6	13.9	7.7	39.4	-19.3	3.3
**Tb. 94-7**	Castiglione	human	adult	N.D.	Tb. 94	25.6	14.3	7.1	38.9	-19.1	3.2
**Tb. 94-10**	Castiglione	human	adult	N.D.	Tb. 94	38.1	13.9	7.7	38.9	-19.2	3.3
**Tb. 94-12**	Castiglione	human	adult	N.D.	Tb. 94	28.3	13.6	6.3	37.9	-18.9	3.2
**Tb. 94-13**	Castiglione	human	adult	N.D.	Tb. 94	10.8	16.8	7.1	47.2	-19.2	3.3
**Tb. 94-8**	Castiglione	human	adult	N.D.	Tb. 94	45.6	14.3	7.1	39.9	-19.1	3.3
**Tb. 94-29**	Castiglione	human	infant (2 yrs)	N.D.	Tb. 94	20.0	14.4	7.5	39.4	-19.0	3.2
**Tb. 94-30**	Castiglione	human	infant (1,5-2,5 yrs)	N.D.	Tb. 94	33.8	12.7	9.3	35.3	-19.1	3.2
**Tb. 94-23**	Castiglione	human	adult	N.D.	Tb. 94	32.1	14.5	8.2	40.6	-19.2	3.3
**Tb 94-1**	Castiglione	human	adult	N.D.	Tb. 94	0.3	-	-	-	-	-
**Tb 94-9**	Castiglione	human	adult	N.D.	Tb. 94	1.0	-	-	-	-	-
**Tb 94-11**	Castiglione	human	adult	N.D.	Tb. 94	5.2	-	-	-	-	-
**Tb 94-25**	Castiglione	human	adult	N.D.	Tb. 94	0.0	-	-	-	-	-
**Tb 94-27**	Castiglione	human	adult	N.D.	Tb. 94	6.2	-	-	-	-	-
**Tb. 96-5**	Castiglione	human	adult	N.D.	Tb. 96	54.6	13.3	6.7	37.2	-19.2	3.3
**Tb. 96-2**	Castiglione	human	adult	N.D.	Tb. 96	39.7	14.5	7.1	40.5	-19.1	3.3
**Tb. 96-3**	Castiglione	human	adult	N.D.	Tb. 96	66.3	15.3	7.4	42.3	-19.7	3.2
**Tb. 96-6**	Castiglione	human	child (10 yrs)	N.D.	Tb. 96	62.6	15.2	6.9	42.3	-19.2	3.2
**Tb. 98-1**	Castiglione	human	adult	N.D.	Tb. 98	12.6	26.8	7.7	75.3	-19.5	3.3
**Tb. 98-4**	Castiglione	human	adult	N.D.	Tb. 98	9.9	12.6	8.3	37.5	-19.5	3.5
**Tb. 98-16 (II)**	Castiglione	human	child (10 yrs)	N.D.	Tb. 98	25.9	14.6	8.1	40.5	-19.3	3.2
**Tb. 98-6**	Castiglione	human	adult	N.D.	Tb. 98	25.7	14.6	7.2	40.6	-19.2	3.2

Table A.2. Summary descriptive statistics for all the specimens available (herbivores, carnivores and omnivores).

Species	Site	δ¹⁵N (‰)				δ¹³C (‰)			
		n	Median	Min	Max	Median	Min	Max	Ref.
Caprines	*Pertuso*	6	3.3	2.9	3.7	-20.5	-21.0	-20.2	This study
	Buco del Diavolo	12	3.9	2.3	5.5	-20.5	-21.5	-19.8	This study
	Ostiglia La Vallona	2	5.5	5.1	5.8	-21.6	-22.0	-21.2	This study
	Ballabio	1	4.7	-	-	-20.3	-	-	7
	Arano di Cellore	3	4.8	2.8	4.9	-19.6	-20.7	-19.2	15
	Olmo di Nogara	1	7.2	-	-	-21.6	-	-	18,19
	Fondo Paviani	4	7.8	6.7	8.3	-18.9	-19.5	-18.4	19
	Grotta Vittorio Vecchi	3	3.4	1.9	4.8	-19.9	-21.5	-19.2	This study
	Grotta dello Scoglietto	4	5.6	4.5	5.8	-21-2	-22.0	-20.2	33
	Grotta Misa	1	5.6	-	-	-21.7	-	-	33
	Grotta Regina Margherita	6	4.4	3.0	4.5	-20.9	-21.3	-19.6	44
	Trinitapoli (Ipogeo dei Bronzi + Madonna di Loreto)	2	6.6	6.2	7.1	-20.5	-20.7	-20.4	This study 18
	Murgia Timone	1	5.6	-	-	-20.7	-	-	50
	Punta di Zambrone	1	5.7	-	-	-14.9	-	-	59
Cattle	*Pertuso*	4	4.0	3.7	5.1	-20.9	-21.3	-19.3	This study
	Buco del Diavolo	1	3.0	-	-	-20.9	-	-	This study
	Ostiglia La Vallona	1	9.0	-	-	-20.7	-	-	This study
	Ballabio	1	4.7	-	-	-20.3	-	-	7
	Arano di Cellore	3	4.2	2.9	5.8	-18.6	-20.7	-18.2	15
	Dossetto di Nogara	1	4.2	-	-	-20.0	-	-	19
	Olmo di Nogara	1	6.5	-	-	-17.8	-	-	18
	Fondo Paviani	5	6.3	5.7	7.2	-18.9	-19.2	-17.6	19
	Mereto	2	4.8	4.5	5.1	-18.0	-20.5	-15.5	18,19
	Gradisca di Codroipo	3	4.2	3.3	5.8	-20.4	-21.8	-19.3	19
	Grotta Vittorio Vecchi	2	5.0	4.6	5.4	-20.3	-20.5	-20.1	This study
	Grotta dello Scoglietto	2	5.2	4.3	6.0	-19.5	-20.7	-18.3	33
	Grotta Misa	2	5.3	4.3	6.3	-20.2	-20.9	-19.4	33
	Grotta Regina Margherita	2	4.4	4.3	4.5	-20.7	-21.6	-19.8	44
	Murgia Timone	2	7.4	7.3	7.4	-19.8	-19.2	-19.1	50
	Punta di Zambrone	3	5.2	4.7	6.0	-17.3	-18.2	-17.0	59
Pig/wild boar	*Pertuso*	5	4.2	3.5	5.2	-21.2	-21.4	-19.5	This study
	Buco del Diavolo	1	3.2	-	-	-20.6	-	-	This study
	Ostiglia La Vallona	1	6.8	-	-	-20.9	-	-	This study
	Ballabio	1	4.3	-	-	-20.7	-	-	7

Species	Site		δ^{15}N (‰)			δ^{13}C (‰)			
		n	Median	Min	Max	Median	Min	Max	Ref.
	Arano di Cellore	5	4.7	3.6	6.1	-20.4	-20.8	-20.2	15
	Dossetto di Nogara	1	5.3	-	-	-20.8	-	-	19
	Fondo Paviani	8	8.1	4.8	9.7	-13.8	-20.6	-11.0	19
	Grotta Vittorio Vecchi	2	3.8	2.9	4.8	-21.3	-21.7	-21.0	This study
	Grotta dello Scoglietto	3	5.6	5.4	5.9	-20.5	-20.9	-20.4	33
	Murgia Timone	1	7.7	-	-	-20.4	-	-	50
	Punta di Zambrone	3	4.3	4.1	5.8	-18.4	-19.5	-16.5	59
**Red deer & roe deer**	*Arano di Cellore*	2	4.3	3.2	5.3	-18.7	-20.0	-17.4	15
	Dossetto di Nogara	1	4.9	-	-	-20.9	-	-	19
	Olmo di Nogara	1	6.3	-	-	-20.9	-	-	19
	Fondo Paviani	1	4.9	-	-	-20.9	-	-	19
	Grotta dello Scoglietto	1	4.2	-	-	-20.8	-	-	33
	Grotta Regina Margherita	1	4.3	-	-	-20.9	-	-	44
	Trinitapoli (Ipogeo dei Bronzi + Madonna di Loreto)	1	7.2	-	-	-20.4	-	-	This study 18
	Punta di Zambrone	4	4.3	3.0	5.4	-19.3	-20.6	-18.0	59
**Lagomorph**	*Buco del Diavolo*	1	1.4	-	-	-21.9	-	-	This study
	Grotta Misa	1	3.5	-	-	-20.2	-	-	33
	Trinitapoli (Ipogeo dei Bronzi + Madonna di Loreto)	1	4.9	-	-	-20.9	-	-	This study 18
**Dog**	*Buco del Diavolo*	2	7.4	6.7	8.2	-19.8	-20.0	-19.6	This study
	Grotta Vittorio Vecchi	1	5.8	-	-	-20.5	-	-	This study
	Grotta Regina Margherita	2	6.7	6.7	6.7	-19.5	-19.6	-19.4	44
	Trinitapoli (Ipogeo dei Bronzi + Madonna di Loreto)	1	8.3	-	-	-18.5	-	-	This study 18
	Murgia Timone	1	8.0	-	-	-19.1	-	-	50
	Punta di Zambrone	3	6.5	5.3	7.6	-16.2	-19.7	-16.1	59
**Fox**	*Grotta dello Scoglietto*	1	7.5	-	-	-20.5	-	-	This study
	Grotta Regina Margherita	2	7.9	7.7	8.0	-19.0	-19.0	-19.0	44
**Horse**	*Mereto*	1	4.5	-	-	-20.2	-	-	19
	Buco del Diavolo	1	3.5	-	-	-20.0	-	-	This study
**Freshwater fish**	*Fondo Paviani (pike)*	2	11.1	12.7		-21.6	-22.7	-20.5	19
	Fondo Paviani (cyprinids)	1	7.9	-	-	-24.7	-	-	19
	Punta di Zambrone	1	5.8	-	-	-18.2	-	-	59

Table A.3. Summary descriptive statistics for all the human isotopic results.

				δ15N			δ13C			
			n	Median	Min	Max	Median	Min	Max	Ref.
**Northern Italy**	***Pertuso***	Human	13	7.2	6.5	7.9	-20.6	-21.0	-20.2	This study
	Buco del Diavolo	Human	9	7.5	6.6	9.1	-18.5	-20.0	-15.0	This study
	Ostiglia La Vallona	Human	5	10.7	10.1	13.3	-13.0	-13.3	-12.6	This study
	Ballabio	Human	22	8.0	7.2	10	-20.4	-20.8	-20.1	7
	Arano di Cellore	Human	54	7.9	6.9	8.9	-20.3	-20.9	-19.7	15
	Bovolone	Human	24	9.1	7.2	13.1	-15.1	-19.6	-10.6	18,19
	Olmo di Nogara	Human	64	9.2	7.5	11.1	-14.8	-17.8	-12.7	18,19
	Dossetto di Nogara	Human	1	8.5	-	-	-13.5	-	-	19
	Sedegliano	Human	2	8.3	8.1	8.4	-17.7	-17.7	-17.6	18
	Mereto	Human	1	7.4	-	-	-20.2	-	-	18
**Central Italy**	***Grotta Vittorio Vecchi***	Human	4	6.4	5.7	6.7	-19.6	-20.1	-19.2	This study
	Grotta dello Scoglietto	Human	11	10.3	9.0	11.5	-20.0	-20.4	-19.5	33
	Grotta Misa	Human	4	8.5	8.1	8.6	-18.2	-19.4	-16.5	33
	Felcetone	Human	12	6.8	6.0	8.8	-19.1	-20.3	-17.3	33
	Grotta Regina Margherita	Human	10	7.2	6.5	9.8	-20.4	-20.7	-19.6	44
**Southern Italy**	***Trinitapoli (Ipogeo dei Bronzi)***	Human	21	8.3	6.5	10	-19.5	-20.0	-18.7	This study<br>50
	Lavello	Human	4	8.3	8.2	9.3	-19.5	-19.6	-19.3	18
	Toppo Daguzzo	Human	21	8.3	6.7	10.2	-19.6	-20.2	-18.9	18,50
	Castiglione	Human	61	7.5	6.3	9.3	-19.2	-19.7	-18.9	This study
	Punta di Zambrone	Human	2	7.7	7.5	7.9	-18.2	-18.2	-18.2	59
	Grotta dell'Antenato	Human	1	8.0	-	-	-19.8	-	-	50
	Grotta della Monaca	Human	6	8.3	7.0	9.5	-19.9	-20.3	-19.6	50

Table A.4
Spearman Rho test for $\delta^{15}N$ and $\delta^{13}C$ with the carbon and nitrogen elemental composition and C/N ranges.

		$\delta^{15}N$ (Rho)	p-value		$\delta^{13}C$ (Rho)	p-value
**Pertuso**	**C/N**	0.417	0.030	**C/N**	-0.018	0.929
	**%N**	-0.317	0.107	**%C**	-0.017	0.933
	**Rdt**	0.512	0.007	**Rdt**	0.190	0.342
**Buco del Diavolo**	**C/N**	-0.072	0.719	**C/N**	-0.279	0.158
	**%N**	0.239	0.230	**%C**	0.484	0.010
	**Rdt**	-0.304	0.124	**Rdt**	-0.019	0.924
**Ostiglia La Vallona**	**C/N**	0.365	0.334	**C/N**	0.0913	0.815
	**%N**	-0.666	0.059	**%C**	-0.55	0.133
	**Rdt**	-0.85	0.006	**Rdt**	-0.73	0.031
**Grotta Vittorio Vecchi**	**C/N**	0.711	0.009	**C/N**	0.301	0.341
	**%N**	-0.126	0.700	**%C**	0.434	0.161
	**Rdt**	-0.084	0.800	**Rdt**	0.168	0.604
**Trinitapoli**	**C/N**	0.666	0.009	**C/N**	-0.191	0.243
	**%N**	-0.222	0.445	**%C**	-0.336	0.512
	**Rdt**	-0.051	0.868	**Rdt**	0.132	0.653
**Castiglione**	**C/N**	0.475	0.000	**C/N**	-0.376	0.003
	**%N**	-0.173	0.182	**%C**	0.021	0.870
	**Rdt**	-0.261	0.042	**Rdt**	0.276	0.031

Table A.5.
Kruskal Wallis test for animals (excluding fox and dogs).

**Northern Italy**		**BAL**	**OLV**	**ARA**	**OdN**	**PER**	**BdD**	**DOSS**	**FP**	**GdC**	**MER**	**H**	**p**
	**$\delta^{15}N$**	3	4	13	5	14	16	3	18	3	3	52.83	0.000
	**$\delta^{13}C$**	3	4	13	5	14	16	3	18	3	3	34.755	0.000
**Central Italy**		**SCO**	**GM**	**GVV**	**GRM**							**H**	**p**
	**$\delta^{15}N$**	10	4	7	9							7.7425	0.0451
	**$\delta^{13}C$**	10	4	7	9							0.36992	0.951
**Southern Italy**		**TRI**	**PdZ**	**MT**								**H**	**p**
	**$\delta^{15}N$**	4	11	4								9.4379	0.002
	**$\delta^{13}C$**	4	11	4								8.4802	0.006
**Early Bronze Age**		**AR**	**BA**	**DOSS**	**ME**	**PERT**	**SCO**					**H**	**p**
	**$\delta^{15}N$**	13	3	3	3	14	10					15.035	0.004
	**$\delta^{13}C$**	13	3	3	3	14	10					10.77	0.041
**Middle Bronze Age**		**FP**	**GM**	**GVV**	**GRM**	**ODN**	**TRI**	**MT**				**H**	**p**
	**$\delta^{15}N$**	18	4	7	9	5	4	4				33.88	0.000
	**$\delta^{13}C$**	18	4	7	9	5	4	4				23.157	0.000
**Recent/ Final Bronze Age**		**OLV**	**BdD**	**GdC**	**PdZ**							**H**	**p**
	**$\delta^{15}N$**	4	16	3	11							13.296	0.000
	**$\delta^{13}C$**	4	16	3	11							19.29	0.000

Table A.6
Kruskal Wallis test for the humans from central Italy

	Scoglietto	GM	Felcetone	GVV	GRM	H	p
**$\delta^{15}N$**	11	4	12	4	10	29.134	0.000
**$\delta^{13}C$**	11	4	12	4	10	22.458	0.000

Exact Wilkoxon-Mann-Whitney test for the humans from central Italy with FRD correction.

**$\delta^{13}C$**	*Gr. Misa*	*Felcetone*	*Gr. Vittorio Vecchi*	*Scoglietto*	*Gr. Regina Margherita*
Gr. Misa	-	Z = -1.6468, p = 0.105 FDR, p = 0.117	Z = -1.8877, p = 0.085 FDR, p = 0.116	Z = 2.8826, p = 0.000 FDR, p = 0.003	Z = -2.8473, p = 0.001 FDR, p = 0.006
Felcetone	Z = -1.6468, p = 0.105 FDR, p = 0.117	-	Z = 1.0392, p = 0.328 FDR, p = 0.328	Z = 1.7237, p = 0.087 FDR, p = 0.116	Z = 3.3859, p = 0.000 FDR, p = 0.002
Gr. Vittorio Vecchi	Z = -1.8877, p = 0.085 FDR, p = 0.116	Z = 1.0392, p = 0.328 FDR, p = 0.328	-	Z = 1.708, p = 0.093 FDR, P = 0.116	Z = -2.4941, p = 0.007 FDR, p = 0.015
Gr. Scoglietto	Z = 2.8826, p = 0.000 FDR, p = 0.003	Z = 1.7237, p = 0.087 FDR, p = 0.116	Z = 1.708, p = 0.093 FDR, p = 0.116	-	Z = -2.6899, p = 0.005 FDR, p = 0.014
Gr. Regina Margherita	Z = -2.8473, p = 0.001 FDR, p = 0.006	Z = 3.3859, p = 0.000 FDR, p = 0.002	Z = -2.4941, p = 0.007 FDR, p = 0.015	Z = -2.6899, p = 0.005 FDR, p = 0.014	-

**$\delta^{15}N$**	*Gr. Misa*	*Felcetone*	*Gr. Vittorio Vecchi*	*Scoglietto*	*Gr. Regina Margherita*
Gr. Misa	-	Z = -2.3075, p = 0.018 FDR, p = 0.036	Z = -2.3374, p = 0.028 FDR, p = 0.028	Z = -2.8774, p = 0.001 FDR, p = 0.002	Z = -2.2804, p = 0.015 FDR, p = 0.028
Felcetone	Z = -2.3075, p = 0.018 FDR, p = 0.036	-	Z = 1.9518, p = 0.056 FDR, p = 0.075	Z = -3.9585, p = 0.000 FDR, p = 0.000	Z = -0.29799, p = 0.782 FDR, p = 0.782
Gr. Vittorio Vecchi	Z = -2.3374, p = 0.028 FDR, p = 0.028	Z = 1.9518, p = 0.056 FDR, p = 0.0754	-	Z = -2.8774, p = 0.001 FDR, p = 0.002	Z = 2.2804, p = 0.022 FDR, p = 0.027
Gr. Scoglietto	Z = -2.8774, p = 0.001 FDR, p = 0.002	Z = -3.9585, p = 0.000 FDR, p = 0.000	Z = -2.8774, p = 0.001 FDR, p = 0.002	-	Z = -3.7407, p = 0.000 FDR, p = 0.000
Gr. Regina Margherita	Z = -2.2804, p = 0.015 FDR, p = 0.028	Z = -0.29799, p = 0.782 FDR, p = 0.782	Z = 2.2804, p = 0.022 FDR, p = 0.027	Z = -3.7407, p = 0.000 FDR, p = 0.000	-

Table A.7
Kruskal Wallis tests for human groups from southern Italy.

	Trinitapoli	TD	Lavello	Gr. della Monaca		H	p
$\delta^{15}N$	21	21	4	6		0.77002	0.868
$\delta^{13}C$	21	21	4	6		8.0623	0.035
	**Trinitapoli**	**TD**	**Lavello**	**Gr. della Monaca**	**Castiglione**	**H**	**p**
$\delta^{15}N$	21	21	4	6	61	33.096	0.000
$\delta^{13}C$	21	21	4	6	61	46.408	0.000

Table A.8. Offsets between the domestic animal (sheep/goat, cattle, pig, horse) and human medians for each Bronze Age site. To be consistent between sites, no wild animals have been included because not all sites have them.

		ARA	BAL	DOSS	MER	SED fauna ME	PERT	SCO
**Early Bronze age**	human	54	22	1	1	2	13	11
	domestic animal	11	3	2	3	3	14	9
	$\Delta^{15}N_{h-f}$	3.2‰	3.7‰	3.7‰	2.9‰	3.8‰	3.5‰	4.7‰
	$\Delta^{13}C_{h-f}$	0.0‰	0.3‰	6.9‰	0.0‰	2.5‰	0.2‰	0.7‰

		ODN	BOV fauna FP[1]	GM	FEL fauna GM	GVV	GRM	TRI	LAV fauna TRI	TD fauna TRI	GdM fauna MT	GdA fauna MT
**Middle Bronze Age**	human	64	24	4	12	4	10	21	4	21	6	1
	domestic animal	4	9	3	3	7	8	2	2	2	4	4
	$\Delta^{15}N_{h-f}$	2.0‰	2.4‰	2.9‰	1.2‰	1.8‰	2.8‰	1.6‰	1.6‰	1.6‰	0.9‰	0.6‰
	$\Delta^{13}C_{h-f}$	5.5‰	3.5‰	2.7‰	1.8‰	0.9‰	0.5‰	1.0‰	1.0‰	0.9‰	0.1‰	0.0‰

		OLV	BdD	PdZ
**Recent/ Final Bronze Age**	human	5	9	2
	domestic animal	4	15	7
	$\Delta^{15}N_{h-f}$	4.4‰	4.0‰	2.5‰
	$\Delta^{13}C_{h-f}$	8.0‰	2.1‰	0.9‰

[1] Given the pigs' exceptional high $\delta^{15}N$ and $\delta^{13}C$ values which overlap with the Bovolone humans they are not included in the calculation of the median animal values. These specimen' values are probably specific only to Fondo Paviani and their inclusion would risk underestimating the animal protein intake of Bovolone humans.

Figure A.4
Map of Early, Middle and Recent-Final Bronze Age sites where archeobotanical remains of common and foxtail millet have been found (the list of the sites is in Supplementary Table B).

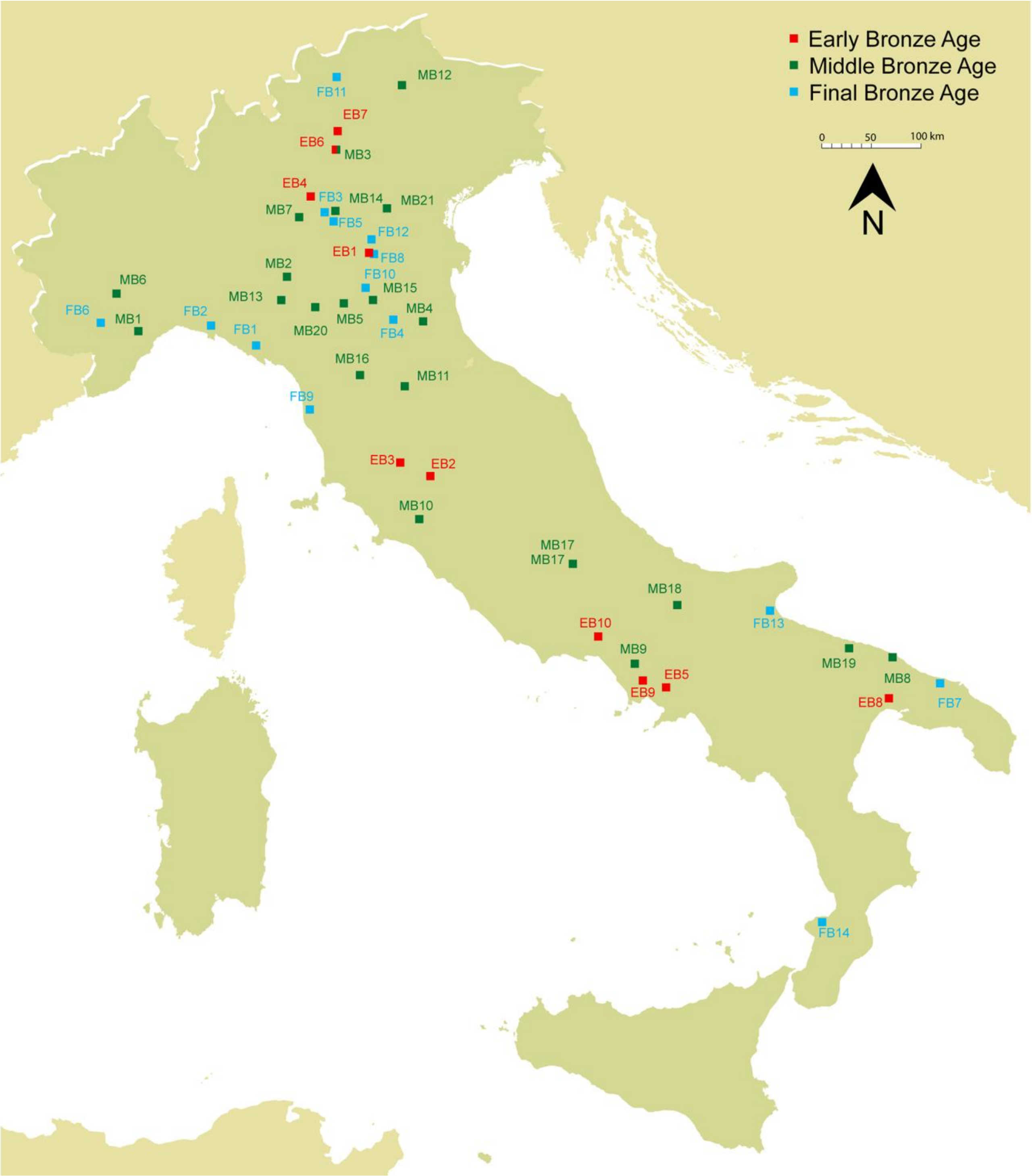

References

1. Varalli, A., Goude, G. & Del Lucchese, A. Dal Neolitico all'Età del Ferro: indagine paleonutrizionale su alcune popolazioni della Liguria attraverso un approccio isotopico. *Archeologia in Liguria* **5**, 11–19 (2015).
2. Del Lucchese, A. *et al.* Il Pertuso. in *Archeologia in Liguria III.1. Scavi e scoperte 1982-1986.* (eds. Melli, P. & Del Lucchese, A.) 169–176 (Soprintendenza archeologica della Liguria, 1987).
3. Del Lucchese, A. & Odetti, G. Nuovi dati sull'antica età del bronzo nella Liguria di Ponente. in *L'Antica età del bronzo in Italia* (ed. Cocchi Genick, D.) 433–440 (Octavo, 1996).
4. Del Lucchese, A. & Ricci, M. Schede, Gli oggetti d'ornamento della Liguria dalla preistoria all'alto medioevo. in *Ori delle Alpi.* (eds. Endrizzi, L. & Marzatico, F.) (Quaderni della sezione archeologica Castello del Buonconsiglio. Monumenti e collezioni provinciali, 1997).
5. Del Lucchese, A. Buco Del Diavolo A Borniga Di Realdo (Triora, Imperia). in *I Liguri. Un antico popolo europeo tra Alpi e Mediterraneo* (eds. De Marinis, R. C. & Spadea, G.) 185–186 (Skira, 2004).
6. Del Lucchese, A. Datazioni radiometriche su resti scheletrici umani dal Buco del Diavolo (Triora) e da altre grotticelle sepolcrali. in *Archeologia in Liguria n.s. I.* (eds. Del Lucchese, A. & Gambaro, L.) 378–379 (De Ferrari, 2008).
7. Masotti, S., Varalli, A., Goude, G., Moggi-Cecchi, J. & Gualdi-Russo, E. A combined analysis of dietary habits in the Bronze Age site of Ballabio (northern Italy). *Archaeological and Anthropological Sciences* **11**, 1029–1047 (2019).
8. Lorenzi, J. & Corti, P. Ballabio (LC) Località Prato della Chiesa. Sepolture dell'Età del Bronzo. 27–28 (2004).
9. Corti, P. Ballabio (LC). Località Prato della Chiesa. Struttura dell'antica età del Bronzo. 117–118 (2005).
10. Lorenzi, J. & Corti, P. Ballabio (LC). Località Prato della Chiesa. Struttura dell'antica età del Bronzo. 82 (2006).
11. Lorenzi, J., Corti, P. & Gaetani, M. Un sito sepolcrale del Bronzo a Ballabio. in *Carta Archeologica della Provincia di Lecco. Aggiornamento.* (ed. Ruffa, M.) 29–52 (Casa editrice Stefanoni, 2009).
12. De Marinis, R. Ostiglia (Mantova). Località " La Vallona". Necropoli birituale dell'età del Bronzo. 33–34 (1986).
13. De Marinis, R. Ostiglia (Mantova). Località "La Vallona". Necropoli birituale dell'età del Bronzo. 24–25 (1987).
14. De Marinis, R. Villaggi e necropoli dell'età del Bronzo nel territorio di Ostiglia. Comune di Ostiglia. 43–60 (1987).
15. Varalli *et al.* Dietary continuity vs. discontinuity in Bronze Age Italy. The isotopic evidence from Arano di Cellore (Illasi, Verona, Italy). *Journal of Archaeological Science: Reports* **7**, 104–113 (2016).
16. Dori, I. & Moggi-Cecchi, J. Brief Communication: An enigmatic enamel alteration on the anterior maxillary teeth in a prehistoric North Italian population. *Am J Phys Anthropol* **154**, 609–14 (2014).
17. Valzolgher, E., Meadows, J., Salzani, P. & Salzani, L. Radiocarbon dating of the Early Bronze Age cemetery at Arano, Verona, northern Italy. *Radiocarbon* **54**, 483–503 (2012).
18. Tafuri, M. A., Craig, O. E. & Canci, A. Stable isotope evidence for the consumption of millet and other plants in Bronze Age Italy. *Am J Phys Anthropol* **139**, 146–53 (2009).
19. Tafuri, M. A. *et al.* Estimating C4 plant consumption in Bronze Age Northeastern Italy through stable carbon and nitrogen isotopes in bone collagen. *International Journal of Osteoarchaeology* **28**, 131–142 (2018).
20. Salzani, L. *La necropoli dell'età del bronzo all'Olmo di Nogara. Memorie del Museo Civico di Storia Naturale di Verona*. (Sezione Scienze dell'uomo. Museo Civico di Storia Naturale, 2005).
21. Salzani, L. *La necropoli dell'età del Bronzo di Bovolone*. (Museo civico di Storia naturale, 2010).
22. Belluzzo, G. & Salzani, L. Dati preliminari di una campagna di scavo dell'abitato dell'età del Bronzo di Dossetto di Nogara. *Annali Benacensi* **13**, 283–288 (1999).
23. Cupitò, M. *et al.* Fondo Paviani (Legnago, Verona): il central place della polity delle Valli Grandi Veronesi nella tarda Età del bronzo. Cronologia, aspetti culturali, evoluzione delle strutture e trasformazioni paleoambientali. in *Preistoria e protostoria del Veneto* (eds. Leonardi, G. & Tiné, V.) (Studi di Preistoria e Protostoria 2, 2015).

24. Borgna, E. & Carozza, S. La prima campagna di scavi nel tumulo di Mereto di Tomba. *Aquileia nostra* **77**, 303–308 (2006).
25. Borgna, E., Corazza, S. & Simeoni, G. Il tumulo di Mereto di Tomba (UD): un monumento funerario cresciuto nel tempo. 34–38 (2013).
26. Cassola Guida, P. & Corazza, S. Dai tumuli ai castellieri: 1500 anni di storia in Friuli (2000- 500 a.C.). *Aquileia Nostra* **76**, 345–360 (2005).
27. Tasca, G., Putzolu, C. & Vicenzutto, D. *Un Castelliere nel Medio Friuli. Gradiscje di Codroipo, 2004-2014*. (Soprintendenza Archeologia del Friuli Venezia Giulia Istituto Regionale per il Patrimonio Culturale, 2015).
28. Formicola, V. & Balestri, M. Il Pertuso. Il materiale scheletrico umano. in *Archeologia in Liguria III.1. Scavi e scoperte 1982-1986.* (eds. Melli, P. & Del Lucchese, A.) 175–176 (Soprintendenza archeologica della Liguria, 1987).
29. Silvestri, S. Nuovi reperti dell'Eneolitico-Bronzo da grotte sepolcrali dell'area ligure-provenzale. Antropologia e comportamento funerario. (Università di Pisa, 2010).
30. Bareschino, A. Condizioni di vita e comportamento funerario in Valle Argentina (Imperia) tra il III e II millennio a.C. (Università degli Studi di Pisa, 2004).
31. Bareschino, A., Del Lucchese, A. & Formicola, V. Condizioni di vita e comportamento funerario dei Liguri tra Bronzo finale ed inizio dell'Età del Ferro: il caso del Buco del Diavolo (Triora). in *I liguri. Un antico popolo europeo tra Alpi e Mediterraneo.* (eds. De Marinis, R. C. & Spadea, G.) 148–151 (Skira, 2004).
32. Accomando, G. Il gruppo umano dell'età del Bronzo Recente della necropoli della Vallona di Ostiglia (Mantova): indagine fenetica e aspetti economico nutrizionali. (Università degli Studi di Pisa, 2002).
33. Varalli, Moggi-Cecchi, J., Moroni, A. & Goude, G. Dietary Variability During Bronze Age in Central Italy: First Results. *International Journal of Osteoarchaeology* **26**, 431–446 (2016).
34. Ceccanti Cocchi, D. La grotta dello Scoglietto (Grosseto). Studio dei materiali conservati al Museo Fiorentino di Preistoria. *Rivista di Scienze Preistoriche* **33**, 187–214 (1978).
35. *Aspetti culturali della media età del bronzo nell'Italia centro-meridionale*. (Octavo, 1995).
36. Ceccanti Cocchi, D. *L'antica età del bronzo nell'Italia centrale: profilo di un'epoca e di un'appropriata strategia metodologica.* (Octavo, 1998).
37. Cocchi Genick, D. & Sarti, L. Bronzo antico e Bronzo medio. in *Atti della XXXIV Riunione Scientifica dell'Istituto Italiano di Preistoria e Protostoria* 91–115 (Istituto Italiano di Preistoria e Protostoria, 2001).
38. Cavanna, C. *La preistoria nelle grotte del Parco Naturale della Maremma*. (Museo di Storia Naturale della Maremma, 2007).
39. Rittatore, F. Lo scavo di un deposito dell'età del bronzo a Grotta Misa in Etruria. in *Atti del 1o Convegno preistorico Italo-svizzero* 85–90 (1949).
40. Rittatore, F. Scoperte di età eneolitica e del bronzo nella Maremma tosco-laziale. *Rivista di Scienze Preistoriche* **6**, 3–33 (1951).
41. Cocchi Genick, D. *Grotta Nuova: la prima unità culturale attorno all'Etruria protostorica*. (Baroni, 2002).
42. Rosini, L. I materiali della Grotta Vittorio Vecchi (Sezze, Latina). in *Atti della XL Riunione Scientifica dell'Istituto Italiano di Preistoria e Protostoria* 695–703 (Istituto Italiano di Preistoria e Protostoria, 2007).
43. Rubini, M., Andreini, L. & Coppa, A. Gli inumati della grotta Vittorio Vecchi di Monte Fulcino (Sezze, Latina; media Eta del Bronzo, 17.-14. sec. a. C.). *Rivista di Antropologia* **68**, 141–163 (1990).
44. Skeates, R. *et al.* Rethinking Collective Burial in Mediterranean Caves: Middle Bronze Age Grotta Regina Margherita, Central Italy. *Journal of Field Archaeology* 1–17 (2021).
45. Angle, M. *et al.* La Grotta Regina Margherita a Collepardo (Frosinone). in *In Lazio e Sabina 6* (ed. Ghini, G.) 381–393 (Soprintendenza per i Beni Archeologici del Lazio, 2010).
46. Rosini, L. Longara. in *Repertorio dei siti protostorici del Lazio. Provincie di Rieti e Latina.* (eds. Belardelli, C. & Pascucci, P.) 63–65 (Centro regionale per la documentazione dei beni culturali e ambientali, 1996).
47. Guidi, A. Recenti ritrovamenti in grotta nel Lazio: un riesame critico del problema dell'utilizzazione delle cavità naturali. *Rassegna di Archeologia* **10**, 427–437 (1992).

48. Costantini, L. & Costantini Biasini, L. Economia agricola del Lazio a sud del Tevere tra Bronzo antico e Bronzo medio. in *Atti della XL riunione scientifica Istituto Italiano di Preistoria e Protostoria* 787–801 (Istituto Italiano di Preistoria e Protostoria, 2007).
49. Carminati, F. L'ipogeo della '"Speranza"' di Lavello (Potenza): lo studio delle sepolture della media eta` del Bronzo. (MA Thesis, Università della Tuscia, Viterbo., 2007).
50. Arena, F., Gualdi-Russo, E., Olsen, J., Philippsen, B. & Mannino, M. A. New data on agro-pastoral diets in southern Italy from the Neolithic to the Bronze Age. *Archaeological and Anthropological Sciences* **12**, 245 (2020).
51. Cipolloni Sampò, M. Le tombe di Toppo Daguzzo (Basilicata Nord-orientale). Considerazioni sulle comunità della media età del Bronzo nel SE italiano. in *Traffici micenei nel Mediterraneo. Problemi storici e documentazione archeologica* (eds. Marazzi, M., Tusa, S. & Vagnetti, L.) 27–39 (Istituto per la storia e l'archeologia della Magna Grecia, 1986).
52. Cipolloni Sampò, M. La tomba 3 dell'acropoli di Toppo Daguzzo (Potenza). Elementi per uno studio preliminare. *Annali dell'Istituto Universitario Orientale, Archeologia e Storia Antica* **8**, 1–36 (1986).
53. Matarese, I. Gli ornamenti della tomba 1 di Murgia Timone (Matera) nel quadro del Bronzo medio in Italia. *Atti XII Incontro di Studi "Preistoria e Protostoria in Etruria: Ornarsi per comunicare agli uomini e agli dei"* **1**, 373–386 (2016).
54. Cremonesi, R. G. Il neolitico e l'età dei metalli. in *Il Museo Nazionale Ridola di Matera* 21–31 (Soprintendenza Archeologica della Basilicata, 1976).
55. Tunzi Sisto, A. M. *Ipogei della Daunia. Culti e riti funerari della media età del Bronzo*. (Grenzi, 1997).
56. *Ipogei della Daunia. Preistoria di un territorio*. (Grenzi, 1999).
57. Pelagatti, P. & Del Campo, M. Abitati siculi: Castiglione. L'abitato castellucciano e le strutture di età arcaica. *Sicilia archeologica* **16**, 31–40 (1971).
58. Pelagatti, P. Castiglione in età preellenica: nota preliminare. in *Camarina 2600 anni dopo la sua fondazione. Nuovi studi sulla città e sul territorio.* (eds. Pelagatti, P., Di Stefano, G. & de Lachenal, L.) 391–395 (Istituto poligrafico e Zecca dello Stato, 2006).
59. Rumolo, A., Forstenpointner, G., Rumolo, P. & Jung, R. Palaeodiet reconstruction inferred by stable isotopes analysis of faunal and human remains at Bronze Age Punta di Zambrone (Calabria, Italy). *International Journal of Osteoarchaeology* **30**, 90–98 (2020).
60. Jung, R., Pacciarelli, M., Zach, B., Klee, M. & Thanheiser, U. Punta di Zambrone (Calabria) – a Bronze Age Harbour Site. First Preliminary Report on the Recent Bronze Age (2011– 2012 Campaigns). *Archaeologia Austriaca* **1**, 53–110 (2015).
61. Arena, F., Larocca, F., Onisto, N. & Gualdi-Russo, E. Il sepolcreto protostorico di Grotta della Monaca in Calabria. Aspetti antropologici. *Annali online dell'Università degli Studi di Ferrara - Museologia Scientifica e Naturalistica* **10**, 74–80 (2014).
62. Larocca, F. *La miniera pre-protostorica di Grotta della Monaca (Sant'Agata di Esaro - Cosenza).* (Centro Regionale di Speleologia "Enzo dei Medici," 2005).
63. Ippolito, F. Late Eneolithic and Early Bronze Age funerary evidence from the Sant'Angelo IV Cave (northeastern Calabria, Italy). *Palaeohistoria* **57/58**, 111–116 (2016).
64. Vanzetti, A. Combinazioni di corredo delle sepolture. in *Ipogei della Daunia. Culti e riti funerari della media età del Bronzo* (ed. Tunzi Sisto, A. M.) 52–60 (Grenzi, 1997).
65. Vanzetti, A. Combinazioni di corredo delle sepolture. in *Ipogei della Daunia. Culti e riti funerari della media età del Bronzo* (ed. Tunzi Sisto, A. M.) 52–60 (Grenzi, 1997).
66. Cenni, S. *et al.* Analisi paleobiologiche degli inumati dell'ipogeo dell'età del Bronzo di Madonna di Loreto (Trinitapoli). *Archivio per l'antropologia e l'etnologia* **129**, 3–82 (1999).
67. Cenni, S. *et al.* Analisi paleobiologiche degli inumati dell'ipogeo dell'età del Bronzo di Madonna di Loreto (Trinitapoli). *Archivio per l'antropologia e l'etnologia* **129**, 3–82 (1999).
68. Di Stefano, G. Castiglione, (s.v.). *Bibliografia Topografica della colonizzazione greca in Italia e nelle Isole Tirreniche* **5**, 130–133 (1987).
69. Mercuri, L. Catiglione di Ragusa: nuovi studi sulla necropoli occidentale. In: (Eds.). Camarina 2600 anni dopo la sua fondazione. Nuovi studi sulla città e sul territorio. Atti del convegno internazionale (7 dicembre 2002/7-9 aprile 2003 Ragusa). Centro studi Felicia. in *Camarina 2600 anni dopo la sua fondazione. Nuovi studi sulla città e sul territorio.* (eds. Pelagatti, P., Di Stefano, G. & de Lachenal, L.) 377–384 (2006).

70. Rovetto, F. Cenni su Castiglione in età preellenica. in *Camarina 2600 anni dopo la sua fondazione. Nuovi studi sulla città e sul territorio.* (eds. Pelagatti, P., Di Stefano, G. & de Lachenal, L.) 395–408 (2006).
71. Mercuri, L. Catiglione di Ragusa: nuovi studi sulla necropoli occidentale. In: (Eds.). Camarina 2600 anni dopo la sua fondazione. Nuovi studi sulla città e sul territorio. Atti del convegno internazionale (7 dicembre 2002/7-9 aprile 2003 Ragusa). Centro studi Felicia. in *Camarina 2600 anni dopo la sua fondazione. Nuovi studi sulla città e sul territorio.* (eds. Pelagatti, P., Di Stefano, G. & de Lachenal, L.) 377–384 (2006).
72. Rovetto, F. Cenni su Castiglione in età preellenica. in *Camarina 2600 anni dopo la sua fondazione. Nuovi studi sulla città e sul territorio.* (eds. Pelagatti, P., Di Stefano, G. & de Lachenal, L.) 395–408 (2006).
73. Facchini, F. I reperti scheletrici della necropoli di Catiglione (Ragusa) (Età del Bronzo). *Archivio per l'antropologia e l'etnologia* **105**, 80–153 (1975).
74. Facchini, F. Sopra una serie di crani dell'Età del Bronzo proveniente dalla necropoli di Castiglione (Ragusa). in *Atti della XV Riunione Scientifica dell'Istituto Italiano di Preistoria e Protostoria* vol. 105 69–85 (Istituto Italiano di Preistoria e Protostoria, 1973).
75. Bocherens, H. & Drucker, D. Trophic level isotopic enrichment of carbon and nitrogen in bone collagen: Case studies from recent and ancient terrestrial ecosystems. *International Journal of Osteoarchaeology* **13**, 46–53 (2003).
76. O'Connell, T. C., Kneale, C. J., Tasevska, N. & Kuhnle, G. G. C. The diet-body offset in human nitrogen isotopic values: A controlled dietary study. *American Journal of Physical Anthropology* **149**, 426–434 (2012).
77. DeNiro, M. J. & Epstein, S. Influence of diet on the distribution of carbon isotopes in animals. *Geochimica et Cosmochimica Acta* **42**, 495–506 (1978).
78. De Grossi Mazzorin, J. lo Sfruttamento delle risrose ittiche in alcuni insediamenti dell'età del Bronzo. in *Atti Preistoria e Protostoria in Etruria V* 257–267 (Centro Studi di Preistoria e Archeologia, 2002).
79. Bonsall, C. *et al.* Radiocarbon and stable isotope evidence of dietary change from the Mesolithic to the Middle Ages in the Iron Gates: new results from Lepenski Vir. *Radiocarbon* **46**, 293–300 (2004).
80. Nehlich, O., Borić, D., Stefanović, S. & Richards, M. P. Sulphur isotope evidence for freshwater fish consumption: a case study from the Danube Gorges, SE Europe. *Journal of Archaeological Science* **37**, 1131–1139 (2010).
81. Richards, M. P., Pettitt, P. B., Stiner, M. C. & Trinkaus, E. Stable isotope evidence for increasing dietary breadth in the European mid-Upper Paleolithic. *Proc Natl Acad Sci U S A* **98**, 6528–32 (2001).
82. Siracusano, G. Le Indagini Archeozoologiche nel Sito Stratificato Di Coppa Nevigata: una visione d'insieme. *Origini* **15**, 201–218 (1991).
83. Tagliacozzo, A., Scali, S. & Cassoli, P. F. La fauna della Grotta Cardini. *Memorie dell'Istituto Italiano di Paleontologia Umana* **4**, 213–257 (1989).
84. Wilkens, B. Gli animali nell'economia degli insediamenti pugliesi dell'età del Bronzo. *Taras* **15**, 491–499 (1995).
85. Mariotti Lippi, M., Pisaneschi, L., Sarti, L., Lari, M. & Moggi-Cecchi, J. Insights into the Copper-Bronze Age diet in Central Italy: Plant microremains in dental calculus from Grotta dello Scoglietto (Southern Tuscany, Italy). *Journal of Archaeological Science: Reports* **15**, 30–39 (2017).
86. Tongiorgi, E. Grano, miglio e fave in un focolare rituale dell'Età del Bronzo a Grotta Misa. *Nuovo Giornale Botanico Italiano* **54**, 804 (1947).
87. Fiorentino, G. Paleoambiente e modalità di sussistenza nell'Età del Bronzo in Puglia. in *Ambra per Agamennone: Indigeni e Micenei tra Adriatico Ionio ed Egeo* (eds. Radina, R. & Recchia, G.) 65–67 (Adda, 2010).
88. Primavera, M., D'Oronzo, C., Muntoni, I. M., Radina, F. & Fiorentino, G. Environment, crops and harvesting strategies during the II millennium BC: Resilience and adaptation in socio-economic systems of Bronze Age communities in Apulia (SE Italy). *Quaternary International* **436**, 83–95 (2017).
89. Miller, D. Stable carbon and nitrogen isotope analysis in Italy and Croatia: Bronze Age food practices across the Adriatic. (University of Rome, La Sapienza, 2018).
90. Di Rita, F. & Magri, D. Holocene drought, deforestation and evergreen vegetation development in the central Mediterranean: a 5500 year record from Lago Alimini Piccolo, Apulia, southeast Italy. *Holocene* **19**, 295–306 (2009).

91. Caroli, I. & Caldara, M. Vegetation history of Lago Battaglia (eastern Gargano coast, Apulia, Italy) during the middle-late Holocene. *Vegetation History and Archaeobotany* **16**, 317–327 (2007).
92. Körner, Ch., Farquhar, G. D. & Wong, S. C. Carbon isotope discrimination by plants follows latitudinal and altitudinal trends. *Oecologia* **88**, 30–40 (1991).
93. Poss, J., Grattan, S. & Suarez, D. Stable carbon isotope discrimination: an indicator of cumulative salinity and boron stress in Eucalyptus camaldulensis. *Tree Physiology* **20**, 1121–1127 (2000).
94. Heaton, T. H. E. Spatial, Species, and Temporal Variations in the 13C/12C Ratios of C3 Plants: Implications for Palaeodiet Studies. *Journal of Archaeological Science* **26**, 637–649 (1999).
95. Stewart, G., Turnbull, M., Schmidt, S. & Erskine, P. 13C natural abundance in plant communities along a rainfall gradient: a biological integrator of water availability. *Australian Journal of Plant Physiology* **22**, 51–55 (1995).
96. Goude, G. & Fontugne, M. Carbon and nitrogen isotopic variability in bone collagen during the Neolithic period: Influence of environmental factors and diet. *Journal of Archaeological Science* **70**, 117–131 (2016).
97. Accomando, G. Il gruppo umano dell'età del Bronzo Recente della necropoli della Vallona di Ostiglia (Mantova): indagine fenetica e aspetti economico nutrizionali. (Università degli Studi di Pisa, 2002).
98. Ferembach, D. Le squelette épicastelnovien de la baume de Montclus (Gard). *Bulletins et Mémoires de la Société d'anthropologie de Paris* **13**, 109–127 (1974).
99. Ferembach, D., Schwidetzky, I. & Stloukal, M. Recommandations pour déterminer l'âge et le sexe sur le squelette. *Bulletins et Mémoires de la Société d'anthropologie de Paris* **6**, 7–45 (1979).
100. Acsádi, G. & Nemeskéri, I. *History of human life span and mortality*. *History of human life span and mortality* (Akadémiai Kiadó, 1970).
101. Black, T. K. Sexual dimorphism in the tooth-crown diameters of the deciduous teeth. *American Journal of Physical Anthropology* **48**, 77–82 (1978).
102. Giles, E. & Elliot, O. Sex determination by discriminant function analysis of crania. *American Journal of Physical Anthropology* **21**, 53–68 (1963).
103. Sauter, M. R. & Privat, F. Sur un noveau procédé métrique de determination sexuelle du bassin osseux. *Bulletin de la Société Suisse d'anthropolgie et d'ethnologie* **31**, 60–84 (1955).
104. Pearson, K. *A study of the long bones of the English skeleton*. (University of London Press, 1917).
105. Steele, D. G. & McKern, T. W. A method for assessment of maximum long bone length and living stature from fragmentary long bones. *American Journal of Physical Anthropology* **31**, 215–227 (1969).
106. Miles, A. E. W. Dentition in the Estimation of Age. *Journal of Dental Research* **42**, 255–263 (1963).
107. Brothwell, D. R. *Digging up bones*. (Oxford University Press, 1981).
108. Lovejoy, C. O., Meindl, R. S., Mensforth, R. P. & Barton, T. J. Multifactorial determination of skeletal age at death: a method with blind tests of its accuracy. *American Journal of Physical Anthropology* **68**, 1–14 (1985).
109. Molnar, S. Human tooth wear, tooth function and cultural variability. *American Journal of Physical Anthropology* **34**, 175–189 (1971).
110. AlQahtani, S. J., Hector, M. P. & Liversidge, H. M. Brief communication: The London atlas of human tooth development and eruption. *American Journal of Physical Anthropology* **142**, 481–490 (2010).
111. Schaefer, M. & Black, S. *Juvenile osteology - A laboratory and field manual*. (Academic Press, 2007).
